# Kidney Tissue Proteome Profiles in Short Versus Long Duration of Delayed Graft Function - A Pilot Study in Donation After Circulatory Death Donors

**DOI:** 10.1016/j.ekir.2024.02.012

**Published:** 2024-02-10

**Authors:** M. Letizia Lo Faro, Kaithlyn Rozenberg, Honglei Huang, Sergei Maslau, Sarah Bonham, Roman Fischer, Benedikt Kessler, Henri Leuvenink, Edward Sharples, Jan H. Lindeman, Rutger Ploeg

**Affiliations:** 1 Nuffield Department of Surgical Sciences, University of Oxford, Oxford, UK; 2 Target Discovery Institute, Nuffield Department of Medicine, University of Oxford, Oxford, UK; 3 University Medical Center Groningen, Groningen, Netherlands; 4 Oxford Transplant Centre, Churchill Hospital, Oxford, UK; 5 Leiden University Medical Centre, Leiden, Netherlands; 6 Oxford Biomedical Research Centre, Oxford, UK

**Keywords:** DCD, DGF, kidney, proteome, organ donation

## Abstract

**Introduction:**

Delayed graft function (DGF) is often defined as the need for dialysis treatment in the first week after a kidney transplantation. This definition, though readily applicable, is generic and unable to distinguish between “types” of DGF or time needed to recover function that may also significantly affect longer-term outcomes. We aimed to profile biological pathways in donation after circulatory death (DCD) kidney donors that correlate with DGF and different DGF durations.

**Methods:**

A total of *N* = 30 DCD kidney biopsies were selected from the UK Quality in Organ Donation (QUOD) biobank and stratified according to DGF duration (immediate function, IF *n* = 10; “short-DGF” (1–6 days), SDGF *n* = 10; “long-DGF” (7–22 days), LDGF *n* = 10). Samples were matched for donor and recipient demographics and analyzed by label-free quantitative (LFQ) proteomics, yielding identification of *N* = 3378 proteins.

**Results:**

Ingenuity pathway analysis (IPA) on differentially abundant proteins showed that SDGF kidneys presented upregulation of stress response pathways, whereas LDGF presented impaired response to stress, compared to IF. LDGF showed extensive metabolic deficits compared to IF and SDGF.

**Conclusion:**

DCD kidneys requiring dialysis only in the first week posttransplant present acute cellular injury at donation, alongside repair pathways upregulation. In contrast, DCD kidneys requiring prolonged dialysis beyond 7 days present minimal metabolic and antioxidant responses, suggesting that current DGF definitions might not be adequate in distinguishing different patterns of injury in donor kidneys contributing to DGF.

## Graphical abstract

**Figure:**
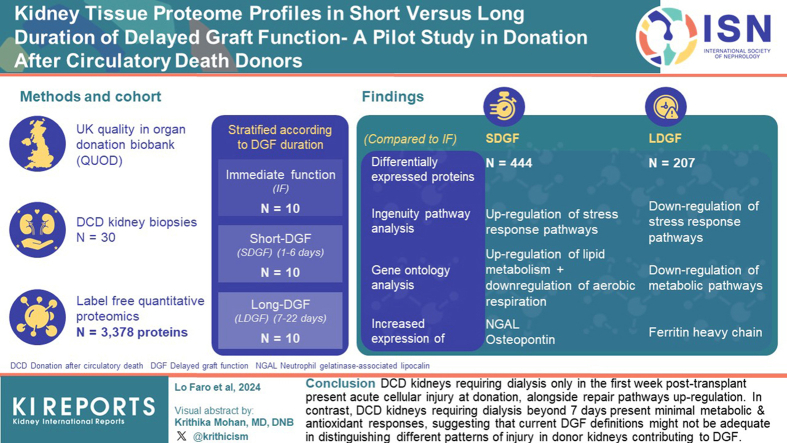

DGF is the manifestation of acute, posttransplant transient failure of the graft to function immediately after kidney transplantation.^1^^,^^2^ DGF associates with prolonged hospitalization, compromised graft function, and impaired long-term graft survival.^3^^,^^4^ DGF is a clinical diagnosis, with different definitions based on changes in posttransplant serum creatinine levels, and/or the need of dialysis treatment immediately posttransplant.^5^^,^^6^ Although the “need for dialysis” is the commonly accepted definition, it remains vague and subjective, often indiscriminately including all dialyses following transplantation (including those due to hyperkalemia and/or fluid overload). The definition also brings no insight into what underpins the mechanism or injury leading to DGF. In fact, posttransplant DGF is the product of preexisting donor susceptibility and events prior to organ procurement, that progress through organ preservation and reperfusion in the recipient. Risk factors that have been associated with DGF are older donor age, donor acute kidney injury (AKI), donor type, prolonged cold ischemia times, recipient biological gender, and high body mass index (BMI).^3^^,^^7^, ^8^, ^9^ DCD donor kidneys have a much higher DGF incidence than donation after brain death kidneys.^10^, ^11^, ^12^ This observation implies a direct and strong association between DCD donation and DGF. We reasoned that this association could provide a unique opportunity to test for possible molecular differences between short and longer duration DGF.^13^ The importance of defining the duration of DGF has been recently highlighted by a UK study in which DGF durations of >14 days for DCD kidneys were associated with an increased risk of death-censored graft failure.^14^

In the present pilot study, we have performed a molecular investigation of DCD donor kidney biopsies obtained immediately after procurement, and by using proteomics, we aimed to elaborate whether short (<7days) and prolonged (≥7days) duration of DGF associate with different biological features (proteins), therefore possibly reflecting different types of injury.

## Methods

### Sample Selection

DCD donor kidney biopsies (16G needle biopsies) were obtained during organ procurement and were provided by the QUOD biobank, according to standardized standard operating procedures. In total, 30 successfully transplanted DCD kidney grafts with known 1-year posttransplant functional outcomes (estimated glomerular filtration rate) were matched for: donor age; biological gender; BMI <30; cold ischemia time ≤18 hours; AKI status (no AKI); and functional warm ischemia time, defined as the interval, after withdrawal of treatment, between the time that the systolic blood pressure is below 50 mm Hg and the start of cold aortic flush-out. Recipients were matched for age, biological gender, and BMI <30. Donor kidney biopsies were further grouped according to kidney function posttransplant and DGF duration, using a 7-day threshold, because the median duration of DGF in the cohort was 7 days, as follows: no DGF (immediate function, IF, *n* = 10), DGF duration up to 6 days (“short” DGF, SDGF, *n* = 10), and DGF between 7 and 22 days (“long” DGF, LDGF, *n* = 10). Wherever possible, we selected donors and concomitant samples where kidney grafts had concordant outcomes (i.e., both kidneys from one donor developed DGF in separate recipients to minimize interference by recipient or preservation factors). In Table 1 and Supplementary Table S1, we summarize donor and recipient demographics. Data about DGF status had been previously identified and collected as part of the UK Transplant Registry Data and data variables were made available retrospectively through a data request to QUOD and National Health Service Blood and Transplant. DGF was defined as the requirement of at least 1 dialysis session in the first week posttransplant. DGF duration was calculated as the difference between the day of transplant and the last documented day of dialysis. This study was conducted under the QUOD Biobank ethical approval.

**Table 1:** Donor and recipient demographics for selected kidney samples

Donor demographics				
Donor type	DCD			
DGF duration	Short DGF (1–6 days) 3 (1–6), *n* = 10 (median, min–max)	Long DGF (7–22 days) 12 (8–16), *n* = 10 (median, min–max)	IF *n* = 10	*P*
Donor age, yr, mean (±SD)	46.20 (±12.14)	43.60 (±13.85)	49.70 (±8.394)	0.5120
Donor biological gender, male/female (% Male)	6/4 (60%)	9/1 (90%)	7/3 (70%)	0.3033
Donor eGFR at procurement, median (min–max)	104.5 (58–210)	98.50 (69–265)	137.0 (68–422)	0.3069
Donor BMI, mean (±SD)	23.19 (±2.537)	24.71 (±3.018)	24.54 (±3.454)	0.4786
F-WIT, min, mean (±SD)	16.40 (±3.688)	20.11 (±4.755) (*n* = 9)	19.10 (±5.782)	0.2357
CIT, h, mean (±SD)	12.30 (±3.305)	11.81 (±3.530)	11.48 (±3.003)	0.8550
Recipient demographics				
Recipient age, yr, mean (±SD)	44.50 (±15.32)	51.90 (±16.18)	48.80 (±13.37)	0.5488
Recipient biological gender, male/female (% Male)	5/5 (50%)	8/2 (80%)	6/4 (60%)	0.3661
Recipient BMI, mean (±SD)	25.91 (±2.776)	25.50 (±2.332)	25.12 (±2.409)	0.7828
Recipient eGFR at 3 mo, mean (±SD)	52.70 (±16.70)	46.70 (±20.11)	54.33 (±22.81) (*n* = 9)	0.6778
Recipient eGFR at 12 mo, mean (±SD)	56.70 (±21.28)	50.60 (±9.22)	53.60 (±22.17)	0.7646

BMI, body mass index; CIT, cold ischemia time; DCD, donation after circulatory death; eGFR, estimated glomerular filtration rate; f-WIT, functional warm ischemia time; max, maximum; min, minimum.

Samples were matched for donor age, donor biological gender, BMI <30, CIT ≤18 hours, AKI status (no AKI), and f-WIT. Recipients of the kidneys were matched for age, biological gender, and BMI <30. eGFR at 3 months and 12 months posttransplant are also reported and are not statistically significantly different between the groups. The 3 groups were compared by 1-way analysis of variance, for normally distributed continuous variables, Kruskal-Wallis test for nonnormally distributed continuous variables and Fisher exact test for categorical variables. Data are reported as mean and SD, except for donor eGFR, which was not normally distributed and reported as median and minimum to maximum.

### Sample Homogenization

Cortical renal tissue biopsies were added to tubes filled with zirconia beads (BioSpec Products, Thistle Scientific, Glasgow, UK) and 250 μl of ice-cold RIPA lysis buffer containing protease inhibitors (cOmplete Mini, Protease Inhibitor Cocktail, Roche, Mannheim, Germany). Samples were spun 3 times at 6500rpm for 40 seconds in a beads-beater homogenizer (Precellys 24, Bertin technologies, Montigny-le-Bretonneux, France) and centrifuged between runs at 18,000g for 1 minute at 4 °C. After homogenization, samples were centrifuged at 10,000g for 10 minutes at 4 °C. The supernatants were collected, and protein content quantified by BCA assay (Pierce Thermo Scientific, Life technologies ltd, Paisley, UK) according to manufacturer’s instructions. To ensure homogenous sample preparation, homogenization for protein extraction took place in 1 round for all samples. Complete homogenization was confirmed by visual inspection of the samples. Protein content for all samples was confirmed by BCA assay and was in range with previous isolations or experiments using similar biopsy samples. The same amount of total protein for each sample was taken forward to protein digestion.

### Filter-Aided Sample Preparation Tryptic Digestion

Proteins were digested to peptides following a filter-aided sample preparation protocol as described^15^ and in the Supplementary Methods).

### Peptide Purification

Peptide digests were purified and desalted on a C18 reverse phase column (Sep-Pak light C18 cartridges, Waters, Dublin, Ireland), according to manufacturer’s instructions and as described in the Supplementary Methods.

### Liquid Chromatography-Tandem Mass Spectrometry (LC-MS/MS) for Protein Identification

Equal amounts (500 ng) of peptide material were analyzed by LC-MS/MS, using nano-UHPLC coupled to a hybrid quadrupole-orbitrap mass spectrometer (Q-Exactive, Thermo Scientific) as described^16^ and in the Supplementary Methods.

### Data Analysis

MS/MS spectra were interpreted using Uniprot human database (undisclosed human 5, version 10/05/2017) and imported to MaxQuant (version 1.5.8.3) for protein quantification. The label-free mass spectrometry intensity data (LFQ) were used to further analyze protein abundance for comparison across groups in Perseus (version 1.6.2.3). LFQs were log2 transformed and further normalized by the median LFQ of each individual sample. Proteins with at least 1 unique peptide and identified in at least 70% of samples in either one of the study groups (SDGF, LDGF, or IF) were brought forward for analysis (*N* = 3378). Statistical comparisons were run between the 3 groups (SDGF vs. IF; LDGF vs. IF, and LDGF vs. SDGF) by *t*-test with permutation-based FDR multiple testing correction (FDR = 0.05). Significant hits included in the canonical pathway analysis performed by IPA were defined as proteins presenting a statistical difference with unadjusted *P* < 0.05. Expression levels (log2 fold change) of all quantified proteins (*N* = 3378) were also analyzed by PANTHER (release 20210224) statistical enrichment test (Mann-Whitney Rank-Sum, U test) to analyze if the expression of biological processes (Gene Ontology Biological Processes) or pathway (Panther or Reactome) significantly differed from the reference distribution of all proteins in the dataset (*P* < 0.05 with FDR < 0.05). The mass spectrometry proteomics data have been deposited to the ProteomeXchange Consortium via the PRIDE^17^ partner repository with dataset identifier PXD038196. Associations between protein expression and DGF duration in days (continuous) were studied by Pearson correlation and corrected for multiple testing (GraphPad Prism version 9.4.1).

### Western Blot Validation of Mass Spectrometry Results

Three proteins identified by LC-MS/MS and presenting significant differences in the comparison between groups were validated by Western blotting in an independent set of *n* = 13 DCD donor kidney biopsies from the QUOD biobank (*n* = 5 SDGF, *n* = 4 LDGF, *n* = 4 IF), in order to provide an orthogonal technique for validation of LC-MS/MS data. The proteins analyzed were NGAL (ab125075, Abcam, Cambridge, UK), Ferritin light chain (FtL, ab109373, Abcam) and RNA-binding protein FUS (ab124923, Abcam). Detailed methods are provided in the Supplementary Methods.

## Results

### Clinical Characteristics

At time of sample selection, 218 DCD kidneys with recorded DGF durations were present in the QUOD biobank (Flowchart 1). Of these kidney recipients, 90% recovered from DGF in the first 22 days posttransplantation and the median DGF duration was 7 days. Therefore, we decided to use 7 days as duration “threshold” and exclude durations longer than 22 days, because they were happening more rarely. Donor and recipient demographics for the *n* = 30 selected kidneys are presented in Table 1 and Supplementary Table S1. Donors in the 3 groups (SDGF, LDGF, and IF) were matched for known DGF risk factors, specifically for donor age and donor biological gender. Interference by other factors (BMI, ischemia times, AKI) was minimized by matching of recipient age and biological gender, as well as by selecting samples with BMI <30, cold ischemia times ≤18 hours, matched functional warm ischemia time <30 minutes, and no AKI. This type of matching was performed to allow us to focus on donor-specific features (proteins) associated with DGF and minimize potential “noise” of other factors.

**Flowchart 1:**
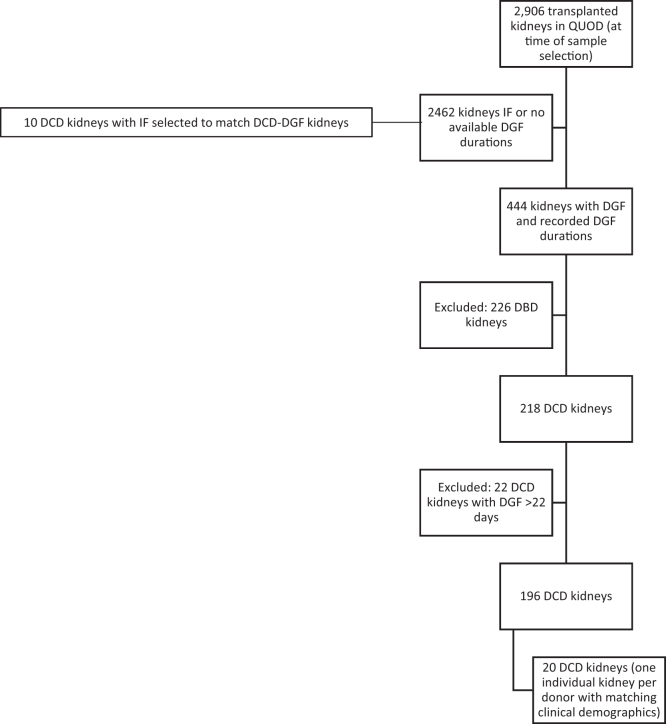
Sample selection process. A total of 2906 transplanted kidneys had biopsy samples available in QUOD at time of sample selection (May 2017). Of these n = 444 had recorded DGF and available DGF durations (date of last dialysis) with n = 218 kidneys from DCD donors. The study focused on 90% of available kidneys which had DGF durations between 1 and 22 days (n = 196). Finally, n = 20 DCD kidneys with DGF were selected so that only 1 kidney per donor was selected; if donors donated 2 kidneys, both had concordant outcomes and kidneys presented matching clinical demographics (Table 1) when comparing n = 10 kidneys with short DGF and n = 10 kidneys with long DGF (shorter and longer than median DGF duration in QUOD biobank). DCD, donation after circulatory death; DGF, delayed graft function; QUOD, UK Quality in Organ Donation.

Estimated glomerular filtration rate values at 3-months and 12-months posttransplant are also reported and were not significantly different between groups (Table 1).

### SDGF and LDGF Kidneys Present Different Proteomic Profiles at Time of Procurement, Compared to Kidneys That Function Immediately Posttransplant

**Figure 1:**
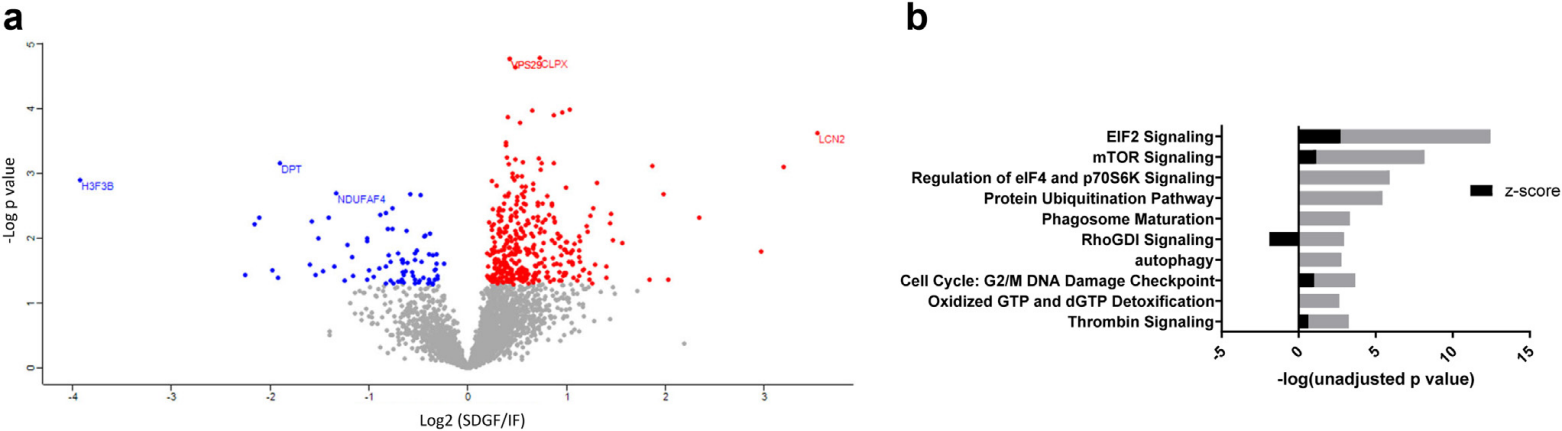
Comparison of proteomic profiles of SDGF and IF kidneys. (a) Scatter plot displaying individual proteins identified in the short DGF (SDGF) and immediate function (IF) groups. *N* = 444 proteins were significantly different (unadjusted *P* < 0.05). In red, proteins significantly upregulated in SDGF compared to IF (*n* = 363) and in blue proteins significantly downregulated in SDGF compared to IF (*n* = 81). X-axis displays the Log2 fold change (SDGF/IF) (*t*-test difference SDGF-IF), and the y-axis displays –Log *P*-value (unadjusted). (b) IPA analysis displaying the top 10 canonical pathways significantly dysregulated in SDGF versus IF. Z-score >0 upregulation; Z-score <0 downregulation in SDGF versus IF. The x axis reports the –log of the unadjusted *P*-values (grey bars) calculated using the right-tailed Fisher exact test in the IPA analysis. CLPX, ATP-dependent Clp protease ATP-binding subunit clpX-like; DGF, delayed graft function; DPT, dermatopontin; H3F3B, histone H3.3; IF, immediate function; IPA, ingenuity pathway analysis; LCN2, neutrophil gelatinase-associated lipocalin; NDUFAF4, NADH dehydrogenase [ubiquinone] 1 alpha sub-complex assembly factor 4; VPS29, vacuolar protein sorting-associated protein 29.

From *n* = 30 DCD procurement kidney biopsies, we identified and quantitated 3378 proteins by LC-MS/MS. When comparing the profiles of SDGF (*n* = 10) to IF (*n* = 10), *n* = 444 differentially expressed proteins (unadjusted *P* < 0.05) were identified (Figure 1a, Supplementary Table S2). IPA revealed that the top 2 pathways predicted to be upregulated in SDGF kidneys were eIF2 and mTOR signaling pathways (Figure 1b), both central to the cellular stress response to ischemia in the kidney.^18^^,^^19^ Additional differentially regulated pathways were the following: protein ubiquitination, phagosome maturation, and autophagy (Figure 1b). It was not possible to predict upregulation or downregulation for these pathways. However, a clear signal was observed for the chaperone-mediated autophagy pathway to be significantly enriched in SDGF, because proteins associated to this pathway (lysosome-associated membrane glycoprotein 2, LAMP2; heat shock protein 70, HSPA4; cathepsin L, CTSL; cathepsin S, CTSS; cathepsin A, CTSA) were all upregulated in SDGF. This finding is similar to what has been reported in kidney ischemia or reperfusion and conditions of nutrients and energy depletion.^20^ In addition, we observed an increased expression of the kidney ischemic damage marker NGAL in SDGF compared to IF (Log2 Fold change (SDGF/IF) = 3.54, *P* = 0.0002). Osteopontin, a glycoprotein expressed in proximal tubules and found elevated in acute and chronic kidney diseases as well as in renal allograft dysfunction, was also increased in SDGF (Log2 Fold change (SDGF/IF) = 2.03, *P* = 0.043) (Supplementary Table S5). Collectively, these results suggest that SDGF kidneys manifest increased damage and upregulation of cell stress-response pathways at time of procurement, when compared to kidneys that functioned immediately (IF).

**Figure 2:**
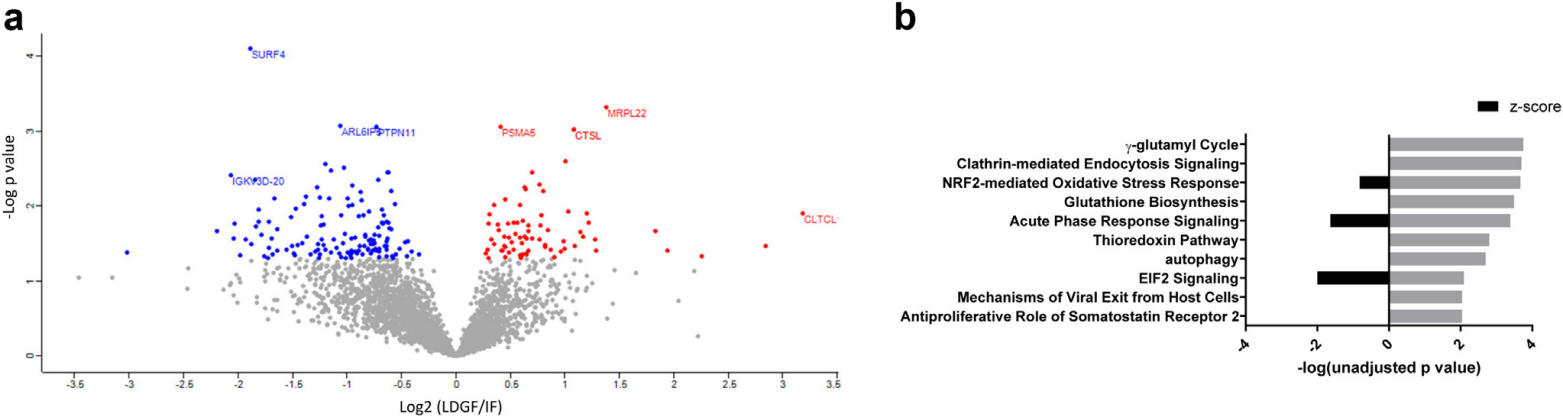
Comparison of proteomic profiles of LDGF and IF kidneys. (a) Scatter plot displaying individual proteins identified in the long DGF (LDGF) and immediate function (IF) groups. *N* = 207 proteins were significantly different (unadjusted *P* < 0.05). In red, proteins significantly upregulated in LDGF compared to IF (*n* = 74) and in blue proteins significantly downregulated in LDGF compared to IF (*n* = 133). X-axis displays the Log2 fold change (LDGF/IF) (*t*- test difference LDGF-IF), and the y-axis displays –Log *P*-value (unadjusted). (b) IPA analysis displaying the top 10 canonical pathways significantly dysregulated in LDGF versus IF. Z-score >0 upregulation; Z-score <0 downregulation in LDGF versus IF. The x axis reports the –log of the unadjusted p values (grey bars) calculated using the right-tailed Fisher's Exact Test in the IPA analysis. ARL6IP5, PRA1 family protein 3; CLTCL, clathrin heavy chain; CTSL, Procathepsin L; DGF, delayed graft function; IGKV3D-20, immunoglobulin kappa variable 3D-20; IPA, ingenuity pathway analysis; MRPL22, 39S ribosomal protein L22; PSMA5, Proteasome subunit alpha type-5; PTPN11, Tyrosine-protein phosphatase non-receptor type 11; SURF4, Surfeit locus protein 4.

Conversely, when comparing the proteomes of LDGF (*n* = 10) to IF kidneys (*n* = 10), we identified *n* = 207 differentially expressed proteins (unadjusted *P* < 0.05) (Figure 2a, Supplementary Table S3). In contrast to the SDGF profile, IPA predicted significant downregulation of stress response pathways, such as Nrf2-mediated oxidative stress response, acute phase response, and eIF2 signaling (Figure 2b). At the individual protein level, we observed increased expression of ferritin heavy chain in LDGF (Log2 Fold change (LDGF/IF) = 1.83, *P* = 0.021). Ferritin heavy chain has a role in kidney injury and is often upregulated in response to high Fe^2+^ levels or proinflammatory cytokines (Supplementary Table S5).

**Figure 3:**
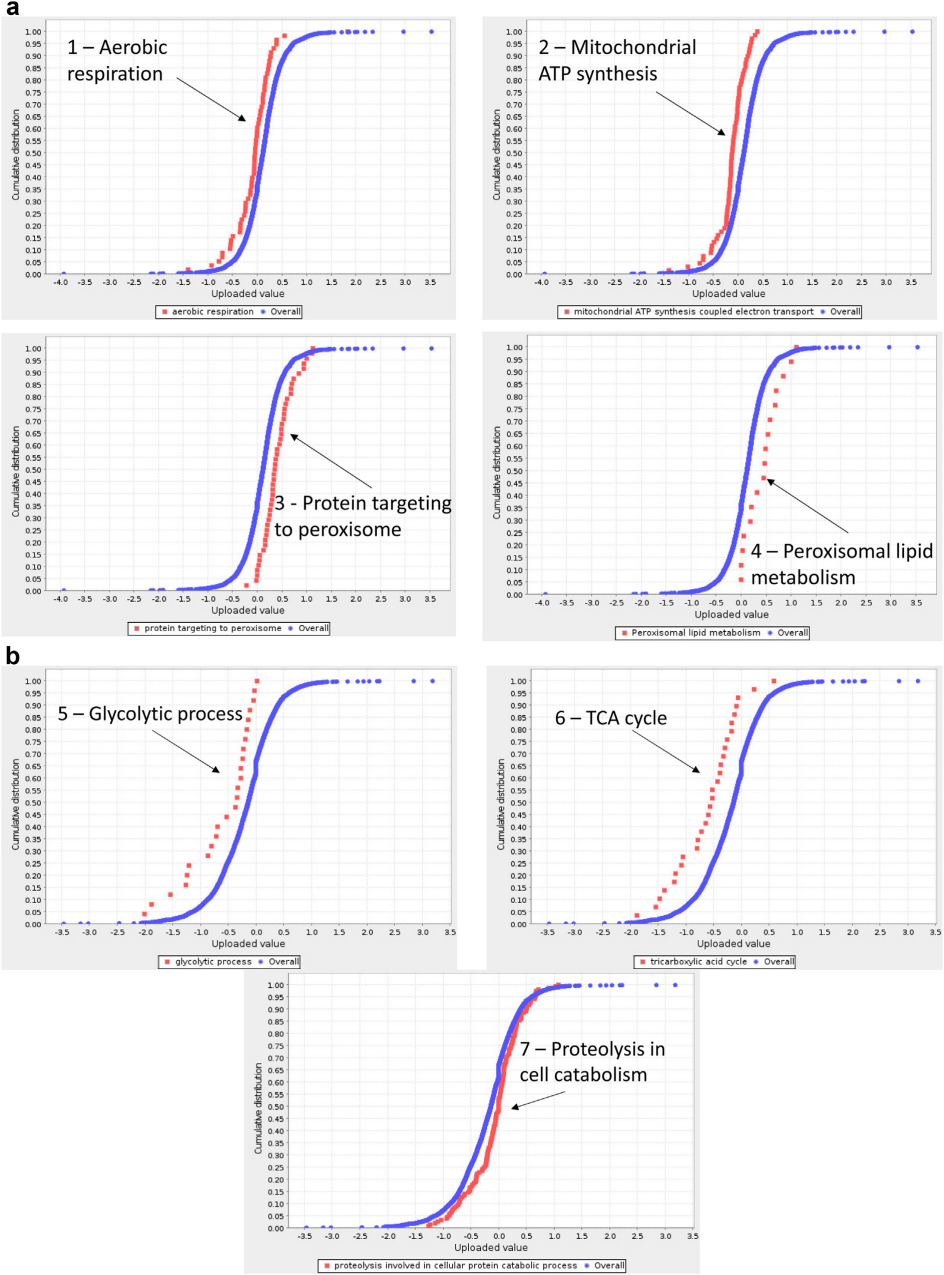
Panther statistical enrichment test of *N* = 3378 proteins and relative fold change for SDGF versus IF (a) and LDGF versus IF (b).The statistical enrichment test (Mann-Whitney Rank-Sum, U test) analyses if the expression of any ontology class (Gene Ontology [GO] Biological Processes or pathway) significantly deviates from the reference distribution of all proteins in the dataset. (a) GO biological processes significantly downregulated (panels 1,2) and upregulated (panels 3,4) in SDGF versus IF. The blue dots represent the overall distribution of log2 (SDGF/IF) for *N* = 3378 proteins in the dataset. The red dots represent individual proteins (and relative expression levels) belonging to specific GO terms (1, aerobic respiration; 2, mitochondrial ATP synthesis; 3, protein targeting to peroxisome and 4, peroxisome lipid metabolism). A shift of the red curve towards the left indicates downregulation and towards the right, upregulation (*P* < 0.05 with FDR multiple testing correction). (b) GO biological processes significantly downregulated (panels 5 and 6) and upregulated (panel 7) in LDGF versus IF. GO terms: 5, glycolytic process; 6, tricarboxylic acid cycle; 7, proteolysis (*P* < 0.05 with FDR multiple testing correction). IF, immediate function; LDGF, long delayed graft function; SDGF, short delayed graft function.

Gene ontology analysis was performed using Panther statistical enrichment test on all quantitated proteins (*N* = 3378) and their relative fold change (log2 (SDGF/IF) and log2 (LDGF/IF), respectively). This analysis showed significant downregulation of aerobic respiration and mitochondrial ATP synthesis in SDGF (compared to IF), with parallel upregulation of protein targeting to peroxisomes and peroxisome lipid metabolism (Figure 3a, Supplementary Figure S1A). LDGF kidneys presented extensive downregulation of metabolic processes, including glycolysis and TCA cycle and increased proteolysis (Figure 3b, Supplementary Figure S1B) compared to IF.

### Comparison of SDGF and LDGF Kidneys Reveals Contrasting Molecular Profiles at Time of Procurement

**Figure 4:**
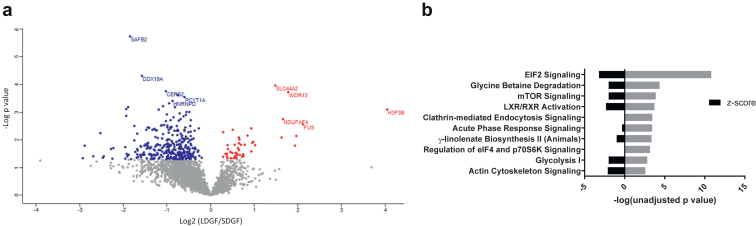
Comparison of proteomic profiles of LDGF and SDGF kidneys. (a) Scatter plot displaying individual proteins identified in the long DGF (LDGF) and short DGF (SDGF) groups. *N* = 367 proteins were significantly different (unadjusted *P* < 0.05). In red, proteins significantly upregulated in LDGF compared to short DGF (SDGF) (*n* = 46) and in blue proteins significantly downregulated in LDGF compared to SDGF (*n* = 321). X-axis displays the Log2 fold change (LDGF/SDGF) (*t*-test difference) and the y axis displays –Log *P*-value (unadjusted). (b) IPA analysis displaying the top 10 canonical pathways significantly dysregulated in LDGF versus SDGF. Z-score >0 upregulation; Z-score <0 downregulation in LDGF versus SDGF. The x axis reports the –log of the unadjusted p values (grey bars) calculated using the right-tailed Fisher's Exact Test in the IPA analysis. CERS2, Ceramide synthase 2; DDX19A, ATP-dependent RNA helicase DDX19A; DGF, delayed graft function; H3F3B, Histone H3.3; HNRNPD, Heterogeneous nuclear ribonucleoprotein D0; IPA, ingenuity pathway analysis; PCYT1A, Choline-phosphate cytidylyltransferase A; SAFB2, Scaffold attachment factor B2; SLC44A2, Choline transporter-like protein 2; WDR13, WD repeat-containing protein 13; NDUFAF4, NADH dehydrogenase [ubiquinone] 1 alpha sub-complex assembly factor 4; FUS, RNA-binding protein FUS.

In addition to the indirect comparison using IF as reference, direct comparison of shorter and longer DGF proteomic profiles revealed contrasting signatures already at procurement (Figure 4a). Three hundred sixty-seven proteins were differentially expressed (unadjusted *P* < 0.05), with the majority having lower expression in LDGF (Figure 4a, Supplementary Table S4). IPA on differentially expressed proteins predicted downregulation of eIF2, mTOR signaling, acute phase response, glycolysis, and osmotic control pathways (glycine betaine degradation) among the top 10 differentially regulated pathways in LDGF versus SDGF (Figure 4b). Among the few proteins that were upregulated in LDGF, we observed increased expression in histone 3.3 and FtL, previously associated with kidney fibrosis and injury, respectively^21^^,^^22^ (Supplementary Table S5). Similarly, Pearson correlation analyses between protein expression and DGF duration as a continuum (days) showed that *n* = 175 proteins were significantly associated with the length of DGF (Pearson correlation *P* < 0.05, following Benjamini-Hochberg correction, FDR 0.05) (Table 2). In Table 2, we present the top 10 proteins found to be associated with DGF duration (ranked by adjusted *P*-value) and relative Pearson *r* coefficients.

**Table 2:** Pearson correlation analyses between normalized LFQ protein expression and DGF duration (days) as a continuum

Protein name	Gene name	Pearson *r*	Benjamini-Hochberg Adjusted P value	Role
Choline transporter-like protein 2	SLC44A2	0.7686	0.009	Involved in choline transport and transmembrane transport. It controls platelet activation and thrombosis
Mitochondrial import receptor subunit TOM34	TOMM34	−0.7855	0.012	Involved in the import of precursor proteins into mitochondria
ATP-dependent Clp protease ATP-binding subunit clpX-like, mitochondrial	CLPX	− 0.7736	0.012	Part of a protease found in mitochondria. This protease is ATP-dependent and targets specific proteins for degradation
Pigment epithelium-derived factor	SERPINF1	−0.7556	0.015	Member of the serpin family with no serine protease inhibitory activity. The encoded protein is secreted strongly inhibits angiogenesis
Dolichol-phosphate mannosyltransferase subunit 3	DPM3	−0.7895	0.016	The protein encoded by this gene is a subunit of dolichol-phosphate mannosyltransferase and acts as a stabilizer subunit.
Scaffold attachment factor B2	SAFB2	−0.8683	0.018	Repressor of estrogen receptor alpha. It is involved in cell cycle regulation, apoptosis, differentiation, the stress response, and regulation of immune genes
Ceramide synthase 2	CERS2	−0.7622	0.018	It plays a role in the regulation of cell growth. It is highly expressed in kidney and liver. It produces very-long-chain ceramide species, considered tissue-protective
Amiloride-sensitive amine oxidase [copper-containing]	AOC1	−0.7193	0.018	A metal-binding membrane glycoprotein that oxidatively deaminates putrescine, histamine, and related compounds
F-box-like/WD repeat-containing protein TBL1XR1	TBL1XR1	−0.7294	0.020	Component of both nuclear receptor corepressor and histone deacetylase 3 complexes. It is required for transcriptional activation
Rho-related GTP-binding protein RhoC	RHOC	−0.7331	0.020	It promotes reorganization of the actin cytoskeleton and regulates cell shape. It is thought to be important in cell locomotion.

DCD, donation after circulatory death; DGF, delayed graft function; LFQ, label-free quantitation.

*N* = 175 proteins were significantly associated with the length of DGF (Pearson correlation *P* < 0.05, following Benjamini-Hochberg correction, FDR 0.05). The table presents the top 10 proteins found to be associated with DGF duration (ranked by adjusted *P*-value) and relative Pearson *r* coefficients. Choline transporter-like protein 2, involved in platelet activation and thrombosis, was found to be increased at time of procurement in DCD grafts with longer DGF duration, whereas other proteins involved in cell cycle regulation, transcription, protein transport, and quality control were all found to be decreased in DCD grafts with long DGF.

**Figure 5:**
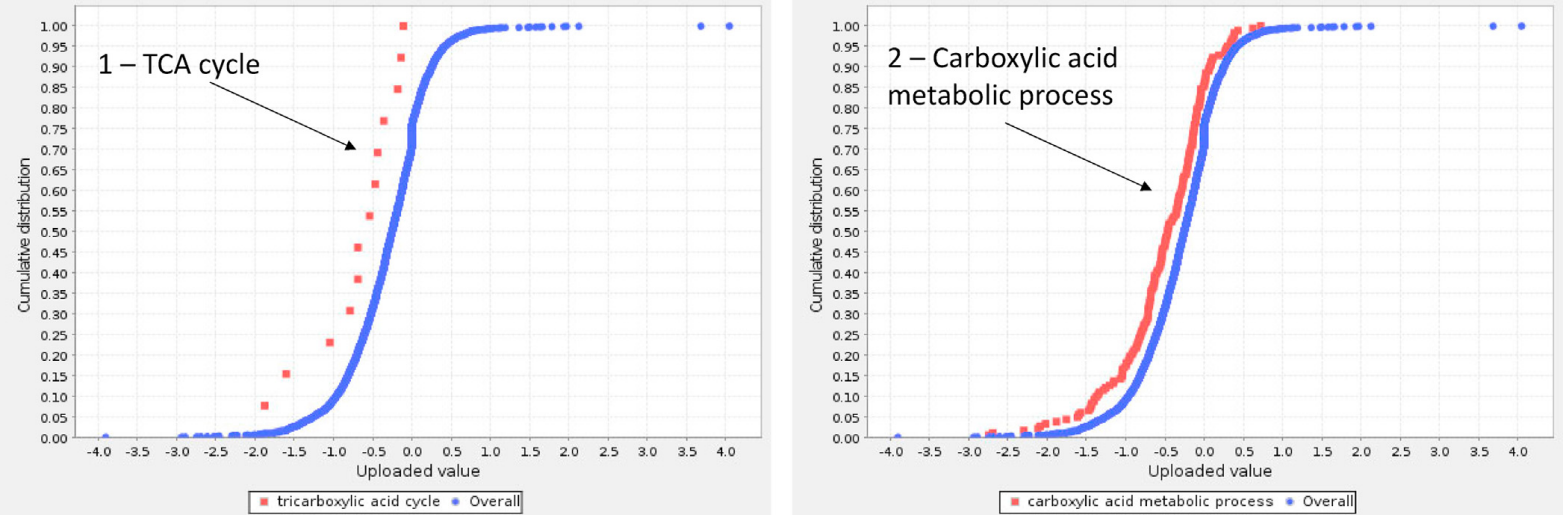
Panther statistical enrichment test of *N* = 3378 proteins and relative fold change for LDGF vs SDGF. The statistical enrichment test (Mann-Whitney Rank-Sum, U test) analyses if the expression of any ontology class (GO Biological Processes or pathway) significantly deviates from the reference distribution of all proteins in the dataset. Panels 1 and 2: GO biological processes significantly downregulated in LDGF vs SDGF. The blue dots represent the overall distribution of log2 (LDGF/SDGF) for *N* = 3378 proteins in the data set. The red dots represent individual proteins (and relative expression levels) belonging to specific GO terms (1, tricarboxylic cycle and 2, carboxylic acid metabolic process). LDGF, long delayed graft function; SDGF, short delayed graft function.

Panther statistical enrichment test on all quantitated proteins (*n* = 3378) and their relative fold change (log2 [LDGF/SDGF]) indicated downregulation of metabolic networks including TCA cycle, carboxylic acid metabolism (Figure 5), and fatty acid oxidation in LDGF (Supplementary Figure S2).

Three differentially expressed proteins (NGAL, FtL, and FUS) were analyzed on an independent set of *n* = 13 DCD kidney procurement biopsies, stratified by kidney outcome as in the proteomics (*n* = 5 SDGF, *n* = 4 LDGF, and *n* = 4 IF). NGAL levels were higher in SDGF compared to IF; however, this did not reach statistical significance (Supplementary Figure S3B). Similarly, FUS presented the same trends as in the mass spectrometry data (decreased in SDGF vs. IF and upregulated in LDGF vs. SDGF); however, this was not statistically significant (Supplementary Figure S3C). No differences were observed in the FtL levels in this set of samples (Supplementary Figure S3D). Given the high interindividual variability observed (high SD), a larger cohort will be needed for further validation of these markers.

## Discussion

DGF in kidney transplantation is a frequent and cumbersome complication that may prolong hospitalization, contribute to undesirable morbidity and rejection, as well as lead to inferior graft survival and outcomes.^3^ Current definitions of DGF are notably heterogeneous.^6^ Registry data imply a strong association between prolonged DGF and inferior transplantation outcomes, showing that the time or duration aspect is important in discriminating between different forms of DGF.^13^^,^^14^ This study aimed to profile the pretransplant (procurement) proteome of DCD donor grafts that developed “short” (<7 days) and “long” DGF (≥7 days) posttransplant, in order to investigate which biological pathways underpin DGF of different durations in DCD donors. For the purpose of this pilot study, we did not include DGF durations >22 days, because the vast majority (90%) of DCD kidneys in the QUOD biobank at time of selection had DGF <22 days. The large collection of transplanted donor kidneys included in the QUOD biobank, which is a unique bioresource of prospectively collected transplant (donor) material linked to clinical outcomes,^23^ allowed us to refine tissue sample selection by matching for DGF-related risk factors and selecting biopsies from donors whose kidney pairs presented similar outcomes, thus attempting to minimize “noise” caused by other donor and recipient factors.

The differences highlighted in the proteomes of grafts with immediate function, compared to DGF of either short or long duration, suggest that donor molecular factors contribute to initiation and extension of DGF, and support the hypothesis that short and longer duration DGF reflect distinct entities. The fact that the duration of DGF might be a clinical factor of importance was also recently highlighted in a published registry data analysis that indicated that prolonged DGF in DCD kidneys is associated with poorer death-censored graft failure.^14^

The focus of this pilot study was to perform an integrated analysis of the tissue proteome in an identifier cohort of well-matched DCD kidneys with opposing DGF outcomes posttransplant, in order to detect molecular changes underpinning DGF. Relative changes in protein expression were integrated through gene ontology analysis in order to map the molecular pathways differentially expressed in grafts with future DGF. The selected 7-day cut-off between short and long duration reflected the median DGF duration for grafts with DGF in the QUOD biobank and is reflective of current clinical definitions of DGF. However, we also found that protein expression was significantly correlated with DGF duration when analyzed as a continuum (Table 2).

From the pathway analysis of short versus prolonged DGF, a seemingly paradoxical picture emerges with comprehensive activation of ischemic stress responses in grafts with short DGF, but not in grafts with longer DGF (e.g., eIF2, mTOR, and autophagy). This is remarkable because, at the same time, LDGF kidneys present increased expression of some proteins that have been previously associated with severe injury in proximal tubules of a mouse model of ischemia reperfusion injury (histone 3.3, FtL, FUS to name a few) and might be related to failed repair.^22^ Absence of a molecular stress response in prolonged DGF might be related to metabolic deficits also observed in these grafts, which render them unable to sustain energy-demanding processes such as protein transcription and translation, modulating cellular repair. Similar observations have been reported in the early postreperfusion phase of living and deceased donors without and with DGF, with the strongest “stress” signal being present in living donors, that is, the “healthier” graft that sustained the least procedural stress.^24^ Exploration of this apparent paradox suggests that the anergy in deceased donors with DGF may reflect a metabolic paralysis caused by depletion of high-energy phosphates. Consequently, insufficient ATP is available to drive the transcriptional machinery required for mRNA synthesis.^24^ Similarly, analysis of perfusate of kidneys preserved by hypothermic machine perfusion has shown that kidneys with DGF release metabolites indicative of metabolic stress and protein degradation whereas kidneys with increased risk of graft failure release metabolites reflective of TCA cycle and alpha-ketoglutarate dysfunction.^25^^,^^26^

In this pilot study, we were able to detect at the proteomic level a metabolic deficit and proteolysis in DGF grafts compared to control kidneys and this was present already at time of procurement. Although short DGF kidneys present downregulation of aerobic respiration and mitochondrial ATP synthesis compared to immediate function, they also show some resilience and parallel upregulation of peroxisome lipid metabolism, as an alternative energy source and as previously shown in a rodent ischemia reperfusion injury model.^27^ This was not the case for long DGF kidneys, which in turn present further downregulation of metabolic pathways (TCA cycle and carboxylic acid metabolism). Therefore, this metabolic deficit might be behind the apparent lack of stress response in kidneys with prolonged DGF and, interestingly, appears to be already present at time of procurement. It suggests that these molecular and metabolic features render the kidneys less able to cope with the ischemia reperfusion-related stress of the preservation and transplant procedure, causing the grafts to take longer time to recover posttransplant. These features were also confirmed when analyzing the associations between protein expression and DGF duration as a continuum. Proteins involved in gene transcription, protein transport, and quality control were all found to be negatively correlated with DGF duration (lower levels of these markers associated with longer DGF).

We selected 13 independent samples to validate the mass spectrometry findings with an orthogonal technique (western blotting). Due to the size of the biopsies and quantities of protein extracted, we could not validate a full pathway; however, we focused on analyzing 3 of the differentially expressed proteins, which showed the highest fold change (at the individual protein level). With the technical validation, we wanted to provide evidence that the proteins are indeed present and measurable in the samples and that the trend is the same as that reported by LC-MS/MS. In the western blotting validation the protein changes had the same trend as in LC-MS/MS, but did not reach statistical significance, which might be due to different factors including the reduced sensitivity of western blotting compared to LC-MS/MS and the high degree of interindividual variability observed (meaning more samples are needed to appropriately power the validation).

A limitation of this study is that tissue sample selection occurred retrospectively and had to rely on clinical data previously collected and recorded in the National Health Service Blood and Transplant data registry, which does not record the reason for dialysis initiation after transplant. A second limitation is that, as with many other “bulk”-omics analyses, we cannot provide spatial information on protein expression and tissue composition. Kidney tissue is quite heterogeneous and by homogenizing the whole biopsy and presenting global proteomic changes, we miss out on more granular information as to what happens in different parts of the kidney. This analysis concerns a first discovery phase pilot study aimed at identifying whether any molecular and biological mechanistic differences are present between different outcome groups. Although this study provides clear mechanistic clues, further studies on a larger validation cohort of samples are needed to confirm the individual protein patterns observed, as well as a putative association between DGF duration and the degree of metabolic dysfunction at time of procurement. In addition, larger studies on unmatched donor cohorts will offer the opportunity to investigate how the kidneys proteomics profiles change in correlation with different clinical variables, including donor variables known to be associated with DGF (e.g., length of warm ischemia time).

In summary, this pilot study shows that DCD kidneys with different durations of DGF after transplantation express contrasting molecular profiles. It also demonstrates that the metabolic status of the donor kidney at time of procurement is important in determining the organ response to ischemia reperfusion injury after preservation and may help identify kidneys that could benefit from different forms of interventions such as normothermic machine perfusion, prior to implantation.

## Disclosure

All the authors declared no competing interests.

## Supplementary Material

**Supplementary Methods.**

Filter-aided sample preparation tryptic digestion.

Peptide purification.

Liquid chromatography-tandem mass spectrometry for protein identification.

Western blot validation of mass spectrometry results.

**Figure S1.** Panther statistical enrichment tests of *N* = 3378 proteins (and relative fold change) for SDGF versus IF (A) and LDGF versus IF (B). LDGF, long delayed graft function; SDGF, short delayed graft function.

**Figure S2.** Panther statistical enrichment tests of *N* = 3378 proteins (and relative fold change) for LDGF versus SDGF.

LDGF, long delayed graft function; SDGF, short delayed graft function.

**Figure S3.** Representative Western blot analysis validation of NGAL, FUS, and Ferritin light chain.

**Table S1**. Additional recipient demographics.

**Table S****2****.** Top 20 proteins significantly upregulated or downregulated in the short DGF (SDGF, *n* = 10) group compared to immediate function (IF, *n* = 10).

**Table S****3****.** Top 20 proteins significantly upregulated or downregulated in the long DGF (LDGF, *n* = 10) group compared to immediate function (IF, *n* = 10).

**Table S****4****.** Top 20 proteins significantly upregulated or downregulated in the long DGF (LDGF, *n* = 10) group compared to short DGF (SDGF, *n* = 10).

**Table S****5****.** Representative differentially expressed proteins in SDGF or LDGF with known reported roles in kidney injury.

STROBE statement checklist.
