# Supplementary material for: Kidney Tissue Proteome Profiles in Short Versus Long Duration of Delayed Graft Function - A Pilot Study in Donation After Circulatory Death Donors
Source: Kidney Int Rep. 2024 Feb 10;9(5):1473–83. doi: 10.1016/j.ekir.2024.02.012 (PMC11068965; doi:10.1016/j.ekir.2024.02.012)
Supplement: Supplementary File (PDF) [file mmc1.pdf]

**Supplementary material**

**Supplementary material and methods**

***2.3. FASP tryptic digestion***

Proteins were digested to peptides following a filter aided sample preparation (FASP) protocol as described in [15] at room temperature. First, all spin filters (Amicon Ultra - 0.5, Ultracel-30 Membrane, 30 kDa, Millipore, Watford, UK) were washed by adding 100μL of milliQ-$H_2O$ and centrifuged at 10,000xg for 10min. 20μg of protein for each sample were added to the filter and centrifuged at 13,000xg for 20min. Samples were reduced by addition of 200mM dithiothreitol (DTT, Sigma Aldrich, Gillingham, UK) and centrifuged at 13,000xg for 20min. Cysteine residues were alkylated by incubation with 200mM iodoacetamide (in 8M urea buffer, Sigma Aldrich) for 30min. After incubation, filters were centrifuged at 10,000xg for 10min and washed with 250μL of 8M urea (in Tris/HCl, pH 8.5) by centrifuging at 13,000xg for 10min. Afterwards, samples were washed twice with 250μL of 0.05M $NH_5CO_3$ and centrifuged at 13,000xg for 10min. Filter units were transferred to fresh collection tubes and trypsin was added at a 1:50 (trypsin:protein) ratio. Trypsin digestion was performed overnight at 37°C with gentle agitation. Upon completion of digestion, samples were briefly vortexed, and filters centrifuged upside down at 5,000xg for 1min to collect samples. Subsequently, 20uL of 1% Formic Acid (FA) were added to stop the trypsin reaction. One hundred (100) μL of 0.5M NaCl were added to the filters and centrifuged upside down at 5,000xg for 1min. Finally, to ensure complete peptide recovery, 100μL of MilliQ-$H_2O$ were added and centrifuged at 10,000xg for 10min.

***2.4. Peptide purification***

Peptide digests were purified and desalted on a C18 reverse phase column (Sep-Pak light C18 cartridges, Waters, Dublin, Ireland) according to manufacturer's instructions. Briefly, the columns were equilibrated with 5mL buffer B (65% Acetonitrile (ACN), 35% MilliQ-$H_2O$, 0.1% formic acid (FA)), followed by 10mL buffer A (98% MilliQ-$H_2O$, 2% ACN, 0.1% FA). Samples were then loaded into the columns, washed with 10mL buffer A and subsequently eluted using 600μL buffer B twice. Peptide fractions were dried by centrifugation under vacuum overnight. Pellets were re-suspended in 40μL of buffer A with final concentration of 0.5 μg/μL, and 1μL was analyzed by LC–MS/MS.

### *2.5. LC-MS/MS for protein identification*

Equal amounts (500ng) of peptide material were analysed by liquid chromatography-tandem mass spectrometry (LC-MS/MS), using nano-UHPLC coupled to a hybrid quadrupole-orbitrap mass spectrometer (Q Exactive, Thermo Scientific) as described in [16]. Briefly, peptides were separated by a C18 Easy spray column (0.75µm x 50cm, Thermo Fisher) at a flow rate of 250 µL/min, using a 60-minute linear gradient from 97% buffer A ($H_2O$ with 0.1% FA) to 40% buffer B (90% ACN with 0.1% FA). After separation, the peptides were ionized by electrospray ionization and injected into the mass spectrometer. Higher energy collisional dissociation (HCD) was induced on the ten most abundant ions per full MS scan to produce MS/MS spectra.

### *2.7 Western blot validation of mass spectrometry results*

An independent set of n=13 samples was selected from the QUOD biobank. Proteins were extracted from the biopsies by homogenization with RIPA buffer and sonication. Protein concentration was determined by BCA assay (Pierce Thermo Scientific, Life technologies ltd, Paisley, UK) and 7ug protein/lane were loaded on a Bis-Tris 4-12% Criterion gel (Bio-Rad), following sample reduction. The following antibodies at the following dilutions were used after transfer of the proteins from the gels to PVDF membranes: NGAL 1:1,000 (ab125075, Abcam, Cambridge, UK), Ferritin light chain 1:20,000 (FtL, ab109373, Abcam), RNA-binding protein FUS 1:500 (ab124923, Abcam) and β-actin 1:16,000 (ab6276, Abcam). Membranes were imaged using a Li-COR Clx Odyssey system and IRDye secondary antibodies (Li-COR, Lincoln, NE, USA). Blots were analyzed with the Image Studio lite software. Statistical analysis was performed with GraphPad Prism (version 9.4.1).

| | | SDGF | LDGF | IF | p |
|---|---|---|---|---|---|
| **Recipient Diabetes Y/N (n)** | | 1/9 | 1/8* | 0/10 | 0.3775 |
| **Dialysis prior to transplant Y/N (n)** | | 9/1 | 8/1* | 8/2 | 0.5167 |
| **Immunosuppression at time of transplant** | MMF (n) | 10 | 7 | 9 | 0.9853 |
| | Prednisolone (n) | 9 | 6 | 10 | |
| | Tacrolimus (n) | 8 | 9 | 10 | |
| | Azathioprine (n) | 1 | 1 | 1 | |
| | Cyclosporin (n) | 1 | 0 | 0 | |
| | Other (not specified) (n) | 10 | 9 | 10 | |

**Table S1. Additional recipient demographics.** For each outcome group (SDGF, LDGF and IF) the table reports the recipient diabetes status (number of recipients with or without diabetes), whether the recipients were on dialysis at time of transplant (yes vs no, n) and the immunosuppression status at time of transplant. Statistical differences between the groups were calculated by Chi-square test for trend. Recipients received combination of drugs for immunosuppression, therefore we tested whether there was a significant difference in the distribution of recipients across drug type. MMF= Mycophenolate Mofetil

*Data not available for n=1 recipient.

**Table S2. Top 20 proteins significantly up– or down-regulated in the short DGF (SDGF, n=10) group compared to immediate function (IF, n=10).** Negative log2 fold change values indicate downregulation in SDGF compared to IF.

| Protein name | Gene name | P value | $Log_2$ Fold change (SDGF/IF) |
|---|---|---|---|
| Dermatopontin | DPT | 6.81E-04 | -1.9 |
| Actin-related protein 2/3 complex subunit 2 | ARPC2 | 3.31E-04 | 0.39 |
| Proteasome subunit alpha type-1 | PSMA1 | 3.64E-04 | 0.39 |
| Proteasome subunit alpha type-3 | PSMA3 | 1.33E-04 | 0.4 |
| Proteasome subunit alpha type-5 | PSMA5 | 5.53E-04 | 0.4 |
| Diablo homolog, mitochondrial | DIABLO | 7.18E-04 | 0.42 |
| Vacuolar protein sorting-associated protein 29 | VPS29 | 1.70E-05 | 0.43 |
| UBX domain-containing protein 4 | UBXN4 | 2.28E-05 | 0.49 |
| Ubiquitin carboxyl-terminal hydrolase isozyme L3 | UCHL3 | 6.02E-04 | 0.49 |
| GTPase-activating protein and VPS9 domain-containing protein 1 | GAPVD1 | 1.63E-04 | 0.53 |
| ATP synthase subunit s-like protein | ATP5SL | 6.53E-04 | 0.56 |
| tRNA (adenine(58)-N(1))-methyltransferase, mitochondrial | TRMT61B | 1.07E-04 | 0.66 |
| Mitochondrial carrier homolog 2 | MTCH2 | 5.77E-04 | 0.72 |
| ATP-dependent Clp protease ATP-binding subunit clpX-like, mitochondrial | CLPX | 1.61E-05 | 0.73 |
| Cathepsin A | CTSA | 6.84E-04 | 0.74 |
| 39S ribosomal protein L22, mitochondrial | MRPL22 | 1.25E-04 | 0.87 |
| Trimethyllysine dioxygenase, mitochondrial | TMLHE | 6.94E-04 | 0.87 |
| Peroxisomal NADH pyrophosphatase NUDT12 | NUDT12 | 1.14E-04 | 0.95 |
| Rho guanine nucleotide exchange factor 12 | ARHGEF12 | 1.04E-04 | 1.03 |
| Neutrophil gelatinase-associated lipocalin | LCN2 | 2.35E-04 | 3.54 |

**Table S3. Top 20 proteins significantly up– or down-regulated in the long DGF (LDGF, n=10) group compared to immediate function (IF, n=10).** Negative log2 fold change values indicate downregulation in LDGF compared to IF.

| Protein name | Gene name | P value | $Log_2$ Fold change (LDGF/IF) |
|---|---|---|---|
| Ig kappa chain V-III region B6 | IGKV3D-20 | 3.88E-03 | -2.06 |
| Surfeit locus protein 4 | SURF4 | 7.85E-05 | -1.89 |
| Guanylate cyclase soluble subunit beta-1 | GUCY1B3 | 4.52E-03 | -1.84 |
| LIM and senescent cell antigen-like-containing domain protein 2 | LIMS2 | 5.61E-03 | -1.27 |
| Costars family protein ABRACL | ABRACL | 2.77E-03 | -1.2 |
| Cat eye syndrome critical region protein 5 | CECR5 | 3.33E-03 | -1.15 |
| PRA1 family protein 3 | ARL6IP5 | 8.41E-04 | -1.06 |
| Golgin subfamily B member 1 | GOLGB1 | 3.11E-03 | -1.03 |
| Microtubule-actin cross-linking factor 1, isoforms 1/2/3/5 | MACF1 | 5.25E-03 | -0.95 |
| Tyrosine-protein phosphatase non-receptor type 11 | PTPN11 | 8.78E-04 | -0.73 |
| Cold shock domain-containing protein E1 | CSDE1 | 4.41E-03 | -0.72 |
| Exportin-1 | XPO1 | 3.58E-03 | -0.63 |
| Signal transducing adapter molecule 2 | STAM2 | 3.58E-03 | -0.62 |
| Proteasome subunit alpha type-5 | PSMA5 | 8.65E-04 | 0.41 |
| Clathrin light chain B | CLTB | 5.54E-03 | 0.63 |
| Glutathione synthetase | GSS | 3.52E-03 | 0.7 |
| Ubiquitin-conjugating enzyme E2 variant 2 | UBE2V2 | 5.10E-03 | 0.76 |
| Regulator of microtubule dynamics protein 1 | RMDN1 | 2.55E-03 | 1 |
| Cathepsin L1 | CTSL | 9.58E-04 | 1.08 |
| 39S ribosomal protein L22, mitochondrial | MRPL22 | 4.81E-04 | 1.38 |

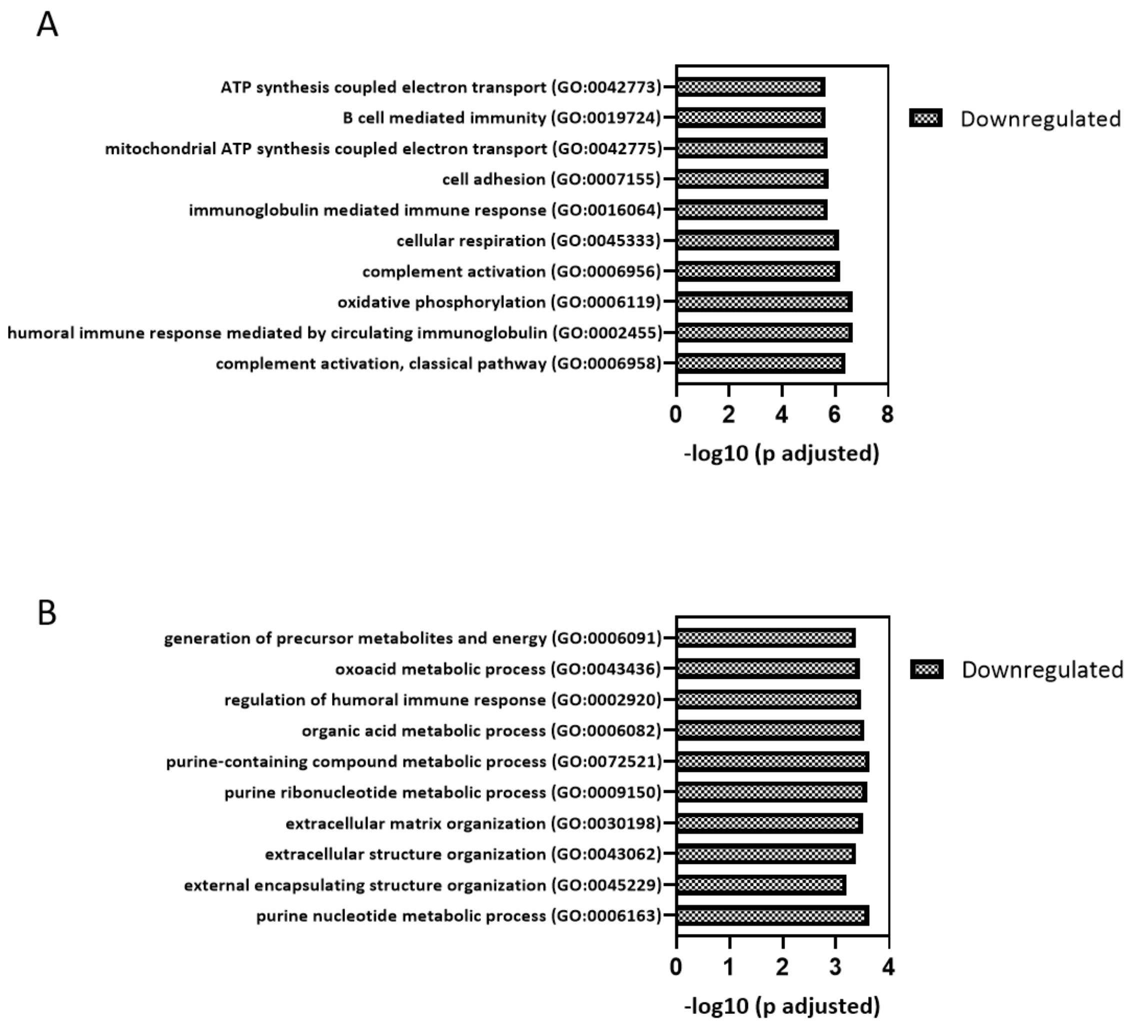


**Figure S1. Panther statistical enrichment tests of n=3,378 proteins (and relative fold change) for SDGF vs IF (A) and LDGF vs IF (B).** The statistical enrichment test (Mann-Whitney Rank-Sum, U test) analyses if the expression of any ontology class (e.g. GO Biological Processes) significantly deviates from the reference distribution of all proteins in the dataset. A) Top 10 GO biological processes significantly regulated in SDGF vs IF. B) Top 10 GO biological processes significantly regulated in LDGF vs IF. GO terms are presented in the y axis, the x axis reports –log10 (adjusted p values) ($p<0.05$ and FDR<0.05 after multiple testing correction).

**Table S4. Top 20 proteins significantly up– or down-regulated in the long DGF (LDGF, n=10) group compared to short DGF (SDGF, n=10).** Negative log2 fold change values indicate downregulation in LDGF compared to SDGF.

| Protein name | Gene name | P value | $Log_2$ Fold change (LDGF/SDGF) |
|---|---|---|---|
| ELKS/Rab6-interacting/CAST family member 1 | ERC1 | 7.51E-04 | -1.94 |
| Surfeit locus protein 4 | SURF4 | 1.34E-03 | -1.94 |
| F-box-like/WD repeat-containing protein TBL1XR1 | TBL1XR1 | 6.45E-04 | -1.89 |
| Scaffold attachment factor B2 | SAFB2 | 1.80E-06 | -1.85 |
| ATP-dependent RNA helicase DDX19A | DDX19A | 4.83E-05 | -1.58 |
| UPF0160 protein MYG1, mitochondrial | MYG1 | 8.00E-04 | -1.19 |
| Solute carrier family 2, facilitated glucose transporter member 1 | SLC2A1 | 6.06E-04 | -1.09 |
| Ceramide synthase 2 | CERS2 | 1.76E-04 | -1.03 |
| Rho-related GTP-binding protein RhoC | RHOC | 1.20E-03 | -1 |
| Signal peptidase complex subunit 2 | SPCS2 | 4.69E-04 | -0.95 |
| Heterogeneous nuclear ribonucleoprotein D0 | HNRNPD | 3.85E-04 | -0.87 |
| Trehalase | TREH | 6.47E-04 | -0.82 |
| UBX domain-containing protein 4 | UBXN4 | 2.39E-04 | -0.75 |
| Choline-phosphate cytidylyltransferase A;Choline-phosphate cytidylyltransferase B | PCYT1A;PCYT1B | 2.83E-04 | -0.6 |
| Zinc transporter SLC39A7 | SLC39A7 | 1.26E-03 | -0.57 |
| Dolichol-phosphate mannosyltransferase subunit 3 | DPM3 | 9.57E-04 | -0.56 |
| 60S ribosomal protein L32 | RPL32 | 9.65E-04 | -0.49 |
| Choline transporter-like protein 2 | SLC44A2 | 1.06E-04 | 1.48 |
| WD repeat-containing protein 13 | WDR13 | 1.86E-04 | 1.78 |
| Histone H3;Histone H3.3;Histone H3.3C | H3F3B;H3F3A;H3F3C | 7.95E-04 | 4.04 |

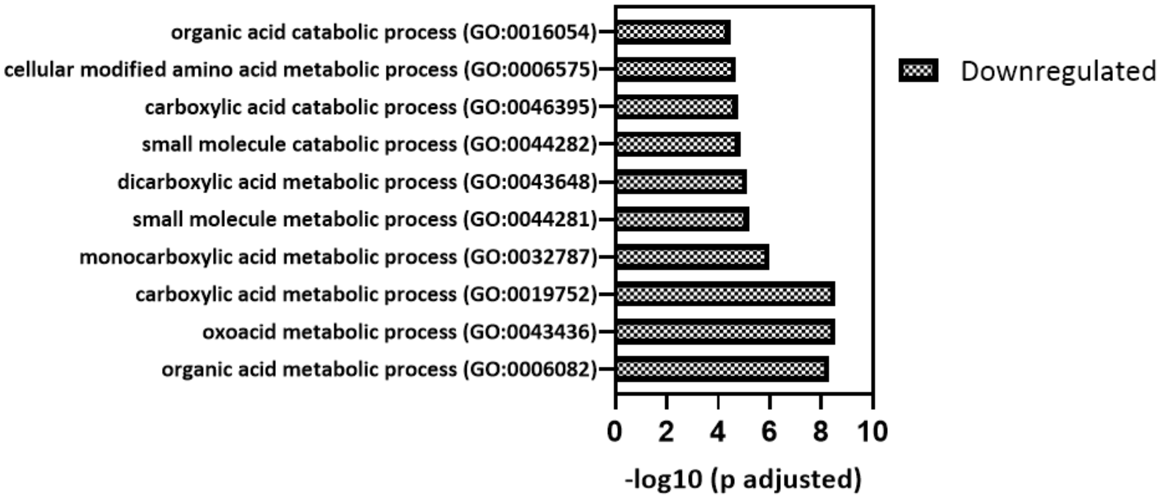


**Figure S2. Panther statistical enrichment tests of n=3,378 proteins (and relative fold change) for LDGF vs SDGF.** The statistical enrichment test (Mann-Whitney Rank-Sum, U test) analyses if the expression of any ontology class (e.g. GO Biological Processes) significantly deviates from the reference distribution of all proteins in the dataset. The figure presents the top 10 GO biological processes significantly regulated in LDGF vs SDGF. GO terms are presented in the y axis, the x axis reports –log10 (adjusted p values) ($p<0.05$ and $FDR<0.05$ after multiple testing correction).

**Table S5. Representative differentially expressed proteins in SDGF or LDGF with known reported roles in kidney injury.** Protein name, localization and reported role are listed alongside $Log_2$ Fold change. Negative log2 fold change values indicate downregulation. Ns= not significant.

| Protein name | $Log_2$ Fold change (SDGF/IF) | $Log_2$ Fold change (LDGF/IF) | $Log_2$ Fold change (LDGF/SDGF) | Localization | Role |
|---|---|---|---|---|---|
| Neutrophil gelatinase-associated lipocalin (NGAL) | 3.54 | ns | -2.89 | Distal Tubules | Iron metabolism. Increased in response to tubular injury |
| Osteopontin (OPN) | 2.03 | ns | ns | Proximal tubule | Regulation of immunity and inflammation, angiogenesis and apoptosis. Expression is elevated in acute and chronic kidney disease and allograft dysfunction |
| Apolipoprotein A-IV (APOA4) | 1.40 | ns | ns | Tubules, blood | Lipid metabolism. Increased plasma and urine levels have been correlated with renal impairment |
| Ferritin heavy chain (FtH) | ns | 1.83 | ns | Proximal tubules | Iron metabolism. Upregulated by pro-inflammatory cytokines |
| Histone H3.3 | -3.9 | ns | 4.04 | Glomeruli and tubules | Transcriptional regulation. Regulates |

| | | | | | | |
|---|---|---|---|---|---|---|
| | | | | | | expression of fibrosis-related genes |
| Ferritin light chain (FtL) | ns | ns | 1.94 | | Proximal tubules | Iron metabolism. Upregulated by pro-inflammatory cytokines |

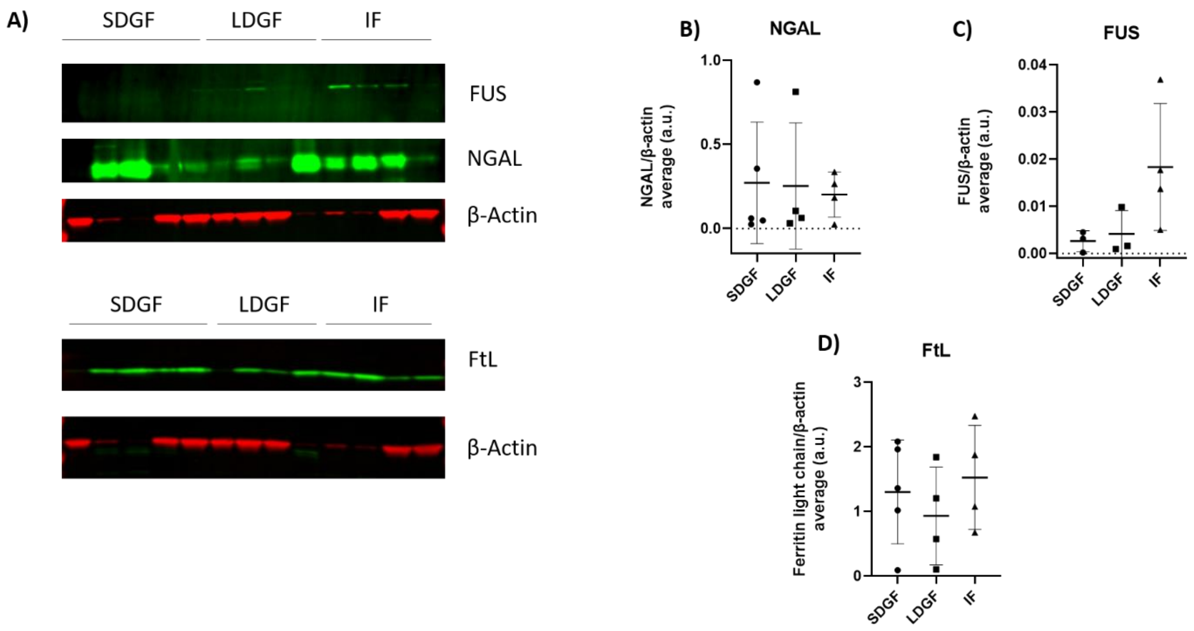


**Figure S3. Representative Western blot analysis validation of NGAL, FUS and Ferritin light chain (FtL).** An independent cohort of n=13 DCD donor kidney samples were selected from the QUOD biobank and stratified according to outcome (n=5 SDGF, n=4 LDGF and n=4 IF). Proteins were extracted and loaded on a gel for western blot analysis (A) of NGAL, FUS and FtL. Intensity of the bands was calculated and normalized to the average β-actin signal for all samples. No significant differences were found between the samples (panels B, C and D), despite trends correlating with the mass spectrometry data.

STROBE Statement—checklist of items that should be included in reports of observational studies

| | Item No. | Recommendation | Page No. | Relevant text from manuscript |
|---|---|---|---|---|
| **Title and abstract** | 1 | (*a*) Indicate the study's design with a commonly used term in the title or the abstract | 1 | Kidney tissue proteome profiling |
| | | (*b*) Provide in the abstract an informative and balanced summary of what was done and what was found | 2 | We aimed to profile biological pathways in DCD kidney donors that correlate with DGF and discriminate between different durations. N=30 DCD kidney biopsies were selected from the UK Quality in Organ Donation biobank and stratified according to DGF duration (immediate function, IF n=10; "short-DGF" (1-6 days), SDGF n=10; "long-DGF" (7-22 days), LDGF n=10). Samples were matched for donor and recipient demographics and analyzed by label-free quantitative proteomics, yielding identification of N=3,378 proteins. Ingenuity Pathway Analysis on differentially abundant proteins showed that SDGF kidneys presented stress response pathways upregulation, while LDGF presented impaired response to stress, compared to IF. LDGF showed extensive |

| | | | | |
|---|---|---|---|---|
| | | | | metabolic deficits compared to IF and SDGF. DCD kidneys requiring dialysis only in the first week post-transplant present acute cellular injury at donation, alongside repair pathways upregulation. In contrast, DCD kidneys requiring dialysis longer beyond 7 days present minimal metabolic and antioxidant responses, suggesting current DGF definitions might not be adequate in distinguishing different patterns of injury in the donor kidneys contributing to DGF. |
| **Introduction** | | | | |
| Background/rationale | 2 | Explain the scientific background and rationale for the investigation being reported | 4-5 | Delayed graft function (DGF) is the manifestation of acute, post-transplant transient failure of the graft to function after kidney transplantation. [1,2] DGF associates with prolonged hospitalization, compromised graft function, and impaired long-term graft survival. [3,4] DGF is a clinical diagnosis, with different definitions based on changes in post-transplant serum creatinine levels, and/or the need of dialysis immediately post-transplant. [5,6] |

| | | | | |
|---|---|---|---|---|
| | | | | Although the 'need for dialysis' is the commonly accepted definition, it remains vague and subjective, often indiscriminately including all dialyses following transplantation (including those due to hyperkalaemia and/or fluid overload). The definition also brings no insight into what underpins the insult leading to DGF. In fact, post-transplant DGF is the product of pre-existing donor susceptibility and events prior to organ procurement, that progress through organ preservation and reperfusion in the recipient. |
| Objectives | 3 | State specific objectives, including any prespecified hypotheses | 4-5 | In the present study we have performed a molecular investigation of DCD donor kidney biopsies obtained immediately after procurement, and by using proteomics, we aimed to elaborate whether short (<7-days) and longer (≥7 days) duration of DGF associate with different biological signatures, and thus may reflect different injuries. |
| **Methods** | | | | |

| | | | | |
|---|---|---|---|---|
| Study design | 4 | Present key elements of study design early in the paper | 5 | DCD donor kidney biopsies (16G needle biopsies) were collected at organ procurement and were provided by the UK Quality in Organ Donation (QUOD) Biobank. In total, 30 successfully transplanted DCD kidney grafts with known 1-year post-transplant functional outcomes (eGFR) were matched for donor age, gender, BMI <30, cold ischemia times (CIT) ≤ 18h, acute kidney injury (AKI) status (no AKI) and functional warm ischemia time (f-WIT), defined as the interval between start of aortic perfusion and the time the systolic blood pressure is below 50 mmHg (after withdrawal of treatment). Recipients were matched for age, gender, and BMI <30. Donor kidney biopsies were further grouped according to kidney function post-transplant and DGF duration, using a 7-day threshold: no DGF (immediate function, IF, n=10); DGF duration up to 6 days (“short” DGF, SDGF, n=10) and DGF between 7 and 22 days (“long” DGF, LDGF, n=10). Wherever possible, we selected |

| | | | | |
|---|---|---|---|---|
| | | | | donors whose kidneys had concordant outcomes |
| Setting | 5 | Describe the setting, locations, and relevant dates, including periods of recruitment, exposure, follow-up, and data collection | 5 | DCD donor kidney biopsies (16G needle biopsies) were collected at organ procurement and were provided by the UK Quality in Organ Donation (QUOD) Biobank. In total, 30 successfully transplanted DCD kidney grafts with known 1-year post-transplant functional outcomes (eGFR) were matched for donor age, gender, BMI <30, cold ischemia times (CIT) ≤ 18h, acute kidney injury (AKI) status (no AKI) and functional warm ischemia time (f-WIT), defined as the interval between start of aortic perfusion and the time the systolic blood pressure is below 50 mmHg (after withdrawal of treatment). Recipients were matched for age, gender, and BMI <30. Donor kidney biopsies were further grouped according to kidney function post-transplant and DGF duration, using a 7-day threshold: no DGF (immediate function, IF, n=10); DGF duration up to 6 days (“short” DGF, SDGF, n=10) and DGF between 7 and 22 days |

| | | | | |
|---|---|---|---|---|
| | | | | ("long" DGF, LDGF, n=10). Wherever possible, we selected donors whose kidneys had concordant outcomes (i.e., both kidneys from one donor developed DGF in separate recipients, to minimize interference by recipient or preservation factors). Table 1 summarizes donor and recipient demographics. DGF status had been previously collected as part of the UK Transplant Registry Data and was made available retrospectively through a data request to QUOD and NHS Blood and Transplant. |
| Participants | 6 | (*a*) *Cohort study*—Give the eligibility criteria, and the sources and methods of selection of participants. Describe methods of follow-up<br>*Case-control study*—Give the eligibility criteria, and the sources and methods of case ascertainment and control selection. Give the rationale for the choice of cases and controls<br>*Cross-sectional study*—Give the eligibility criteria, and the sources and methods of selection of participants | 5-6 | Cohorts of samples were selected from previously collected kidney biopsy samples stored in QUOD biobank. Selection of samples was retrospective. Sample selection criteria is described DCD donor kidney biopsies (16G needle biopsies) were collected at organ procurement and were provided by the UK Quality in Organ Donation (QUOD) Biobank. In total, 30 successfully transplanted DCD kidney grafts with known 1-year |

post-transplant functional outcomes (eGFR) were matched for donor age, gender, BMI <30, cold ischemia times (CIT) ≤ 18h, acute kidney injury (AKI) status (no AKI) and functional warm ischemia time (f-WIT), defined as the interval between start of aortic perfusion and the time the systolic blood pressure is below 50 mmHg (after withdrawal of treatment). Recipients were matched for age, gender, and BMI <30. Donor kidney biopsies were further grouped according to kidney function post-transplant and DGF duration, using a 7-day threshold: no DGF (immediate function, IF, n=10); DGF duration up to 6 days ("short" DGF, SDGF, n=10) and DGF between 7 and 22 days ("long" DGF, LDGF, n=10). Wherever possible, we selected donors whose kidneys had concordant outcomes (i.e., both kidneys from one donor developed DGF in separate recipients, to minimize interference by recipient or preservation factors). Table 1 summarizes donor and recipient demographics. DGF

| | | | |
|---|---|---|---|
| | | | status had been previously collected as part of the UK Transplant Registry Data and was made available retrospectively through a data request to QUOD and NHS Blood and Transplant. DGF was defined as the requirement of at least one dialysis session in the first week post-transplant. DGF duration was calculated as the difference between the day of transplant and the last documented day of dialysis. This study was conducted under the QUOD Biobank ethical approval. |
| | (*b*) *Cohort study*—For matched studies, give matching criteria and number of exposed and unexposed<br><br>*Case-control study*—For matched studies, give matching criteria and the number of controls per case | 5-6 | In total, 30 successfully transplanted DCD kidney grafts with known 1-year post-transplant functional outcomes (eGFR) were matched for donor age, gender, BMI <30, cold ischemia times (CIT) ≤ 18h, acute kidney injury (AKI) status (no AKI) and functional warm ischemia time (f-WIT), defined as the interval between start of aortic perfusion and the time the systolic blood pressure is below 50 mmHg (after withdrawal of treatment). Recipients were matched for |

| | | | | |
|---|---|---|---|---|
| | | | | age, gender, and BMI <30. Donor kidney biopsies were further grouped according to kidney function post-transplant and DGF duration, using a 7-day threshold: no DGF (immediate function, IF, n=10); DGF duration up to 6 days ("short" DGF, SDGF, n=10) and DGF between 7 and 22 days ("long" DGF, LDGF, n=10). |
| Variables | 7 | Clearly define all outcomes, exposures, predictors, potential confounders, and effect modifiers. Give diagnostic criteria, if applicable | 5-6 | DCD donor kidney biopsies (16G needle biopsies) were collected at organ procurement and were provided by the UK Quality in Organ Donation (QUOD) Biobank. In total, 30 successfully transplanted DCD kidney grafts with known 1-year post-transplant functional outcomes (eGFR) were matched for donor age, gender, BMI <30, cold ischemia times (CIT) ≤ 18h, acute kidney injury (AKI) status (no AKI) and functional warm ischemia time (f-WIT), defined as the interval between start of aortic perfusion and the time the systolic blood pressure is below 50 mmHg (after withdrawal of treatment). Recipients were matched for age, gender, and |

BMI <30. Donor kidney biopsies were further grouped according to kidney function post-transplant and DGF duration, using a 7-day threshold: no DGF (immediate function, IF, n=10); DGF duration up to 6 days ("short" DGF, SDGF, n=10) and DGF between 7 and 22 days ("long" DGF, LDGF, n=10). Wherever possible, we selected donors whose kidneys had concordant outcomes (i.e., both kidneys from one donor developed DGF in separate recipients, to minimize interference by recipient or preservation factors). Table 1 summarizes donor and recipient demographics. DGF status had been previously collected as part of the UK Transplant Registry Data and was made available retrospectively through a data request to QUOD and NHS Blood and Transplant. DGF was defined as the requirement of at least one dialysis session in the first week post-transplant. DGF duration was calculated as the difference between the day

| | | | | |
|---|---|---|---|---|
| | | | | of transplant and the last documented day of dialysis |
| Data sources/ measurement | 8* | For each variable of interest, give sources of data and details of methods of assessment (measurement). Describe comparability of assessment methods if there is more than one group | 6-7-8 | Cortical renal tissue biopsies were added to tubes filled with zirconia beads (BioSpec Products, Thistle Scientific, Glasgow, UK) and 250µL of ice-cold RIPA lysis buffer containing protease inhibitors (cOmplete™ Mini, Protease Inhibitor Cocktail, Roche, Mannheim, Germany). Samples were spun three times at 6,500rpm for 40 seconds in a beads-beater homogeniser (Precellys 24, Bertin technologies, Montigny-le-Bretonneux, France) and centrifuged between runs at 18,000xg for 1min at 4°C. After homogenisation, samples were centrifuged at 10,000xg for 10min at 4°C. The supernatants were collected and protein content quantified by BCA assay (Pierce Thermo Scientific, Life technologies ltd, Paisley, UK) according to manufacturer's instructions.<br>2.3. FASP tryptic digestion<br>Proteins were digested to peptides following a filter-aided sample preparation (FASP) |

protocol as described in 15 and in the supplementary material.

2.4. Peptide purification

Peptide digests were purified and desalted on a C18 reverse phase column (Sep-Pak light C18 cartridges, Waters, Dublin, Ireland), according to manufacturer's instructions and as described in the supplementary material.

2.5. LC-MS/MS for protein identification

Equal amounts (500ng) of peptide material were analyzed by liquid chromatography-tandem mass spectrometry (LC-MS/MS), using nano-UHPLC coupled to a hybrid quadrupole-orbitrap mass spectrometer (Q-Exactive, Thermo Scientific) as described in 16 and in the supplementary material.

2.6. Data analysis

MS/MS spectra were interpreted using Uniprot human database (undisclosed human 5, version 10/05/2017) and imported to MaxQuant

(version 1.5.8.3) for protein quantification. The label-free mass spectrometry intensity data (LFQ) were used to further analyze protein abundance for comparison across groups in Perseus (version 1.6.2.3). LFQs were log2 transformed and further normalized by the median LFQ of each individual sample. Proteins with at least one unique peptide and identified in at least 70% of samples in either one of the study groups (SDGF, LDGF, IF) were brought forward for analysis (N=3,378). Statistical comparisons were run between the three groups (SDGF vs IF; LDGF vs IF and LDGF vs SDGF) by Student's t-test with permutation-based FDR multiple testing correction (FDR=0.05). Significant hits included in the canonical pathway analysis performed by Ingenuity Pathway Analysis (IPA) were defined as proteins presenting a statistical difference with unadjusted $p<0.05$. Expression levels (log2 fold change) of all quantified proteins (N=3,378) were also analyzed by PANTHER (release

20210224) statistical enrichment test (Mann-Whitney Rank-Sum, U test) to analyze if the expression of any ontology class (GO Biological Processes) or pathway (Panther or Reactome) significantly deviated from the reference distribution of all proteins in the dataset ($p<0.05$ with $FDR<0.05$). The mass spectrometry proteomics data have been deposited to the ProteomeXchange Consortium via the PRIDE 17 partner repository with dataset identifier PXD038196. Associations between protein expression (LFQ) and DGF duration in days were studied by Pearson correlation (GraphPad Prism version 9.4.1).

2.7. Western blot validation of mass spectrometry results

Three proteins identified by LC-MS/MS and presenting significant differences in the comparison between groups were validated by Western blotting in an independent set of n=13 DCD donor kidney biopsies from the QUOD

| | | | | |
|---|---|---|---|---|
| | | | | biobank (n=5 SDGF, n=4 LDGF, n=4 IF). The proteins analyzed were NGAL (ab125075, Abcam, Cambridge, UK), Ferritin light chain (FtL, ab109373, Abcam) and RNA-binding protein FUS (ab124923, Abcam). Detailed methods are provided in the supporting information |
| Bias | 9 | Describe any efforts to address potential sources of bias | 5 | Wherever possible, we selected donors whose kidneys had concordant outcomes (i.e., both kidneys from one donor developed DGF in separate recipients, to minimize interference by recipient or preservation factors). |
| Study size | 10 | Explain how the study size was arrived at | 5 | Pilot study of n=30 kidney biopsies |

Continued on next page

| | | | | |
|---|---|---|---|---|
| Quantitative variables | 11 | Explain how quantitative variables were handled in the analyses. If applicable, describe which groupings were chosen and why | 7 | 2.6. Data analysis<br><br>MS/MS spectra were interpreted using Uniprot human database (undisclosed human 5, version 10/05/2017) and imported to MaxQuant (version 1.5.8.3) for protein quantification. The label-free mass spectrometry intensity data (LFQ) were used to further analyze protein abundance for comparison across groups in Perseus (version 1.6.2.3). LFQs were log2 transformed and further normalized by the median LFQ of each individual sample. Proteins with at least one unique peptide and identified in at least 70% of samples in either one of the study groups (SDGF, LDGF, IF) were brought forward for analysis (N=3,378). Statistical comparisons were run between the three groups (SDGF vs IF; LDGF vs IF and LDGF vs SDGF) by Student's t-test with permutation-based FDR multiple testing correction (FDR=0.05). Significant hits included in the canonical pathway analysis performed by Ingenuity Pathway Analysis (IPA) were defined as proteins presenting a statistical difference with unadjusted $p<0.05$. Expression |

| | | | | |
|---|---|---|---|---|
| | | | | levels (log2 fold change) of all quantified proteins (N=3,378) were also analyzed by PANTHER (release 20210224) statistical enrichment test (Mann-Whitney Rank-Sum, U test) to analyze if the expression of any ontology class (GO Biological Processes) or pathway (Panther or Reactome) significantly deviated from the reference distribution of all proteins in the dataset (p<0.05 with FDR<0.05). The mass spectrometry proteomics data have been deposited to the ProteomeXchange Consortium via the PRIDE 17 partner repository with dataset identifier PXD038196. Associations between protein expression (LFQ) and DGF duration in days were studied by Pearson correlation (GraphPad Prism version 9.4.1). |
| Statistical methods | 12 | (*a*) Describe all statistical methods, including those used to control for confounding | 7 | Statistical comparisons were run between the three groups (SDGF vs IF; LDGF vs IF and LDGF vs SDGF) by Student's t-test with permutation-based FDR multiple testing correction (FDR=0.05). Significant hits included in the canonical pathway analysis performed by Ingenuity Pathway Analysis (IPA) were defined as proteins presenting a statistical difference |

| | | | | |
|---|---|---|---|---|
| | | | | with unadjusted p<0.05. Expression levels (log2 fold change) of all quantified proteins (N=3,378) were also analyzed by PANTHER (release 20210224) statistical enrichment test (Mann-Whitney Rank-Sum, U test) to analyze if the expression of any ontology class (GO Biological Processes) or pathway (Panther or Reactome) significantly deviated from the reference distribution of all proteins in the dataset (p<0.05 with FDR<0.05). |
| | | (*b*) Describe any methods used to examine subgroups and interactions | 7 | As above |
| | | (*c*) Explain how missing data were addressed | | N/A |
| | | (*d*) *Cohort study*—If applicable, explain how loss to follow-up was addressed<br><br>*Case-control study*—If applicable, explain how matching of cases and controls was addressed<br><br>*Cross-sectional study*—If applicable, describe analytical methods taking account of sampling strategy | | N/A |
| | | (*e*) Describe any sensitivity analyses | | N/A |
| **Results** | | | | |
| Participants | 13* | (a) Report numbers of individuals at each stage of study—eg numbers potentially eligible, examined for eligibility, confirmed eligible, included in the study, completing follow-up, and analysed | 8 | 3.1 Clinical characteristics<br><br>At time of sample selection, 218 DCD kidneys with recorded DGF durations were present in the QUOD biobank (Flowchart). 90% of these kidney recipients recovered from DGF in the first 22 days post- |

| | | | | |
|---|---|---|---|---|
| | | | | transplantation. Donor and recipient demographics for the n=30 selected kidneys are presented in table 1. Donors in the three groups (short DGF, long DGF and IF) were matched for known DGF risk factors, specifically donor age and donor gender. Interference by other factors (BMI, ischemia times, AKI) was minimized by matching of recipient age and gender, as well as by selecting samples with BMI <30, CIT ≤ 18h, matched f-WIT<30 min and no AKI. |
| | | (b) Give reasons for non-participation at each stage | | N/A |
| | | (c) Consider use of a flow diagram | Ref page 8 | Flow diagram included |
| Descriptive data | 14* | (a) Give characteristics of study participants (eg demographic, clinical, social) and information on exposures and potential confounders | 8 + Table 1 | 3.1 Clinical characteristics<br><br>At time of sample selection, 218 DCD kidneys with recorded DGF durations were present in the QUOD biobank (Flowchart). 90% of these kidney recipients recovered from DGF in the first 22 days post-transplantation. Donor and recipient demographics for the n=30 selected kidneys are presented in table 1. Donors in the three groups (short DGF, long DGF and IF) were matched for known DGF risk factors, specifically donor age and donor gender. |

| | | | | |
|---|---|---|---|---|
| | | | | Interference by other factors (BMI, ischemia times, AKI) was minimized by matching of recipient age and gender, as well as by selecting samples with BMI <30, CIT ≤ 18h, matched f-WIT<30 min and no AKI.<br><br>eGFR values at 3- and 12-months post-transplant are also reported and were not statistically significantly different between groups (Table 1). |
| | | (b) Indicate number of participants with missing data for each variable of interest | | Table 1 |
| | | (c) *Cohort study*—Summarise follow-up time (eg, average and total amount) | | Table 1 |
| Outcome data | 15* | *Cohort study*—Report numbers of outcome events or summary measures over time | | Table 1 + sample selection |
| | | *Case-control study*—Report numbers in each exposure category, or summary measures of exposure | | *N/A* |
| | | *Cross-sectional study*—Report numbers of outcome events or summary measures | | *N/A* |
| Main results | 16 | (*a*) Give unadjusted estimates and, if applicable, confounder-adjusted estimates and their precision (eg, 95% confidence interval). Make clear which confounders were adjusted for and why they were included | 8-11 | Proteomics data are reported with unadjusted and adjusted values |
| | | (*b*) Report category boundaries when continuous variables were categorized | | N/A |
| | | (*c*) If relevant, consider translating estimates of relative risk into absolute risk for a meaningful time period | | N/A |

Continued on next page

| | | | | |
|---|---|---|---|---|
| Other analyses | 17 | Report other analyses done—eg analyses of subgroups and interactions, and sensitivity analyses | | N/A |
| **Discussion** | | | | |
| Key results | 18 | Summarise key results with reference to study objectives | 12-14 | This study aimed to profile the pre-transplant (procurement) proteome of DCD donor grafts that developed “short” (<7 days) and “long” DGF (≥7 days) post-transplant, in order to investigate which biological pathways underpin DGF of different duration in DCD donors. For the purpose of this study we did not include DGF durations >22 days, as the vast majority (90%) of DCD kidneys in the QUOD biobank at time of selection had DGF<22 days. The large collection of transplanted donor kidneys included in the QUOD biobank, a unique bio-resource of prospectively collected transplant (donor) material linked to clinical outcomes 19, allowed for a refined sample selection by matching for DGF-related risk factors and selecting biopsies from donors whose kidney pairs presented similar outcomes.<br><br>The differences highlighted in the procurement proteomes of grafts with immediate function, compared to DGF of either short or long duration, suggest that donor |

molecular factors contribute to initiation and extension of DGF and also support the hypothesis that short and longer duration DGF reflect distinct entities. The fact that the duration of DGF might be a clinical factor of importance was also recently highlighted in a published registry data analysis that indicated that prolonged DGF in DCD kidneys is associated with poorer death-censored graft failure. 14

The focus of this study was to perform an integrated analysis of the tissue proteome of well-matched DCD kidneys with opposing DGF outcomes post-transplant, in order to identify molecular changes underpinning DGF. Relative protein expressions were integrated through gene ontology analysis in order to map the molecular pathways differentially expressed in grafts with future DGF. The selected 7-day cut-off between short and long duration reflected the median DGF duration for grafts with DGF in the QUOD biobank and is also reflective of current clinical definitions of DGF. However, we also analyzed whether protein

expression was significantly correlated with DGF duration as a continuum.

From the pathway analysis of short vs long DGF, a seemingly paradoxical picture emerges with comprehensive activation of stress responses in grafts with short DGF, but not in grafts with longer DGF. Absence of a molecular stress response in long DGF might be ascribed to metabolic deficiencies, also observed in these grafts, which render them unable to sustain energy-demanding processes, including protein transcription and translation. Similar observations have been reported in the early post-reperfusion phase of living and deceased donors without and with DGF, with the strongest 'stress' signal in living donors, i.e. the "healthier" graft that sustained the least procedural stress. 20 Exploration of this apparent paradox suggested that the anergy in deceased donors with DGF reflected a metabolic paralysis caused by depletion of high-energy phosphates. Consequently, insufficient ATP is available to drive the transcriptional machinery required for mRNA synthesis. 20

Similarly, analysis of perfusate of kidneys preserved by hypothermic machine perfusion has shown that kidneys with DGF release metabolites indicative of metabolic stress and protein degradation. 21

At the proteomic level we were also able to detect metabolic deficit and proteolysis in DGF grafts compared to control kidneys and this was present already at time of procurement. While short DGF kidneys present downregulation of aerobic respiration and mitochondrial ATP synthesis compared to immediate function, they also show some resilience and parallel upregulation of peroxisome lipid metabolism, as an alternative energy source and as previously shown in a rodent IRI model. 22 This was not the case for long DGF kidneys, which in turn present further downregulation of metabolic pathways (TCA cycle and carboxylic acid metabolism). Hence this metabolic deficit might be behind the apparent lack of stress response in the kidneys with longer DGF and, interestingly, is already present at time of procurement. It appears that these molecular and metabolic features render the

| | | | | |
|---|---|---|---|---|
| | | | | kidneys less able to cope with the IR-related stress of the preservation and transplant procedure, causing the grafts to take longer time to recover post-transplant. These features were also confirmed when analyzing the associations between protein expression and DGF duration as a continuum. Proteins involved in gene transcription, protein transport and quality control were all found to be negatively correlated with DGF duration (lower levels of these markers associated with longer DGF). |
| Limitations | 19 | Discuss limitations of the study, taking into account sources of potential bias or imprecision. Discuss both direction and magnitude of any potential bias | 14-15 | A limitation of this study is that sample selection occurred retrospectively and had to rely on clinical data previously collected and recorded in the NHSBT data registry, which does not record the reason for dialysis initiation after transplant. A second limitation is that, as with many other "bulk" -omics analyses, we cannot provide spatial information on protein expression and tissue composition. Kidney tissue is quite heterogenous and by homogenizing the whole biopsy and presenting global proteomic changes we miss out on more granular information as to |

| | | | | |
|---|---|---|---|---|
| | | | | what happens in different parts of the kidney. This study represents a first discovery phase study aimed at identifying whether any molecular and biological mechanistic differences are present between different outcome groups. While this study provides clear mechanistic clues, further studies on a larger validation cohort of samples will be needed to confirm the individual protein patterns observed, as well as a putative association between DGF duration and the degree of metabolic inertia at time of procurement. |
| Interpretation | 20 | Give a cautious overall interpretation of results considering objectives, limitations, multiplicity of analyses, results from similar studies, and other relevant evidence | 12-15 | Delayed graft function in kidney transplantation is a frequent and cumbersome complication that may prolong hospitalization, contribute to undesirable morbidity, rejection as well as lead to inferior graft survival and outcomes. 3 Current definitions of DGF are notably heterogeneous. 6 Registry data imply a strong association between prolonged DGF and transplantation outcomes, showing that the time/duration aspect is important in discriminating between different forms of DGF. 13,14 This study aimed to profile the pre-transplant |

(procurement) proteome of DCD donor grafts that developed "short" (<7 days) and "long" DGF (≥7 days) post-transplant, in order to investigate which biological pathways underpin DGF of different duration in DCD donors. For the purpose of this study we did not include DGF durations >22 days, as the vast majority (90%) of DCD kidneys in the QUOD biobank at time of selection had DGF<22 days. The large collection of transplanted donor kidneys included in the QUOD biobank, a unique bio-resource of prospectively collected transplant (donor) material linked to clinical outcomes 19, allowed for a refined sample selection by matching for DGF-related risk factors and selecting biopsies from donors whose kidney pairs presented similar outcomes.

The differences highlighted in the procurement proteomes of grafts with immediate function, compared to DGF of either short or long duration, suggest that donor molecular factors contribute to initiation and extension of DGF and also support the hypothesis that short and longer duration DGF

reflect distinct entities. The fact that the duration of DGF might be a clinical factor of importance was also recently highlighted in a published registry data analysis that indicated that prolonged DGF in DCD kidneys is associated with poorer death-censored graft failure. 14

The focus of this study was to perform an integrated analysis of the tissue proteome of well-matched DCD kidneys with opposing DGF outcomes post-transplant, in order to identify molecular changes underpinning DGF. Relative protein expressions were integrated through gene ontology analysis in order to map the molecular pathways differentially expressed in grafts with future DGF. The selected 7-day cut-off between short and long duration reflected the median DGF duration for grafts with DGF in the QUOD biobank and is also reflective of current clinical definitions of DGF. However, we also analyzed whether protein expression was significantly correlated with DGF duration as a continuum.

From the pathway analysis of short vs long DGF, a seemingly paradoxical picture emerges with comprehensive activation of stress responses in grafts with short DGF, but not in grafts with longer DGF. Absence of a molecular stress response in long DGF might be ascribed to metabolic deficiencies, also observed in these grafts, which render them unable to sustain energy-demanding processes, including protein transcription and translation. Similar observations have been reported in the early post-reperfusion phase of living and deceased donors without and with DGF, with the strongest 'stress' signal in living donors, i.e. the "healthier" graft that sustained the least procedural stress. 20 Exploration of this apparent paradox suggested that the anergy in deceased donors with DGF reflected a metabolic paralysis caused by depletion of high-energy phosphates. Consequently, insufficient ATP is available to drive the transcriptional machinery required for mRNA synthesis. 20 Similarly, analysis of perfusate of kidneys preserved by hypothermic machine perfusion has shown that kidneys with DGF release

metabolites indicative of metabolic stress and protein degradation. 21

At the proteomic level we were also able to detect metabolic deficit and proteolysis in DGF grafts compared to control kidneys and this was present already at time of procurement. While short DGF kidneys present downregulation of aerobic respiration and mitochondrial ATP synthesis compared to immediate function, they also show some resilience and parallel upregulation of peroxisome lipid metabolism, as an alternative energy source and as previously shown in a rodent IRI model. 22 This was not the case for long DGF kidneys, which in turn present further downregulation of metabolic pathways (TCA cycle and carboxylic acid metabolism). Hence this metabolic deficit might be behind the apparent lack of stress response in the kidneys with longer DGF and, interestingly, is already present at time of procurement. It appears that these molecular and metabolic features render the kidneys less able to cope with the IR-related stress of the preservation and transplant procedure, causing the grafts to

take longer time to recover post-transplant. These features were also confirmed when analyzing the associations between protein expression and DGF duration as a continuum. Proteins involved in gene transcription, protein transport and quality control were all found to be negatively correlated with DGF duration (lower levels of these markers associated with longer DGF).

A limitation of this study is that sample selection occurred retrospectively and had to rely on clinical data previously collected and recorded in the NHSBT data registry, which does not record the reason for dialysis initiation after transplant. A second limitation is that, as with many other “bulk” -omics analyses, we cannot provide spatial information on protein expression and tissue composition. Kidney tissue is quite heterogenous and by homogenizing the whole biopsy and presenting global proteomic changes we miss out on more granular information as to what happens in different parts of the kidney. This study represents a first discovery phase study aimed at identifying whether any molecular

| | | | | |
|---|---|---|---|---|
| | | | | and biological mechanistic differences are present between different outcome groups. While this study provides clear mechanistic clues, further studies on a larger validation cohort of samples will be needed to confirm the individual protein patterns observed, as well as a putative association between DGF duration and the degree of metabolic inertia at time of procurement.<br><br>In summary, this study shows that DCD kidneys with different durations of DGF after transplantation express contrasting molecular profiles. It also demonstrates that the metabolic status of the donor kidney at time of procurement is important in determining the organ response to IRI after preservation, and may help identify kidneys that could benefit from different forms of interventions such as normothermic machine perfusion, prior to implantation. |
| Generalisability | 21 | Discuss the generalisability (external validity) of the study results | 14-15 | This study represents a first discovery phase study aimed at identifying whether any molecular and biological mechanistic differences are present between |

| | | | | |
|---|---|---|---|---|
| | | | | different outcome groups. While this study provides clear mechanistic clues, further studies on a larger validation cohort of samples will be needed to confirm the individual protein patterns observed, as well as a putative association between DGF duration and the degree of metabolic inertia at time of procurement.<br><br>In summary, this study shows that DCD kidneys with different durations of DGF after transplantation express contrasting molecular profiles. It also demonstrates that the metabolic status of the donor kidney at time of procurement is important in determining the organ response to IRI after preservation, and may help identify kidneys that could benefit from different forms of interventions such as normothermic machine perfusion, prior to implantation. |
| **Other information** | | | | |
| Funding | 22 | Give the source of funding and the role of the funders for the present study and, if applicable, for the original study on which the present article is based | 15 | The present work was funded by a grant from NHS Blood and Transplant "WP15-07 Organ Conditioning Unit: repair injury and |

| | |
|---|---|
| | recover organs for transplantation”. |

*Give information separately for cases and controls in case-control studies and, if applicable, for exposed and unexposed groups in cohort and cross-sectional studies.

**Note:** An Explanation and Elaboration article discusses each checklist item and gives methodological background and published examples of transparent reporting. The STROBE checklist is best used in conjunction with this article (freely available on the Web sites of PLoS Medicine at http://www.plosmedicine.org/, Annals of Internal Medicine at http://www.annals.org/, and Epidemiology at http://www.epidem.com/). Information on the STROBE Initiative is available at www.strobe-statement.org.
